# Validation of the kidney failure risk equation for end-stage kidney disease in Southeast Asia

**DOI:** 10.1186/s12882-019-1643-0

**Published:** 2019-12-04

**Authors:** Yeli Wang, Francis Ngoc Hoang Long Nguyen, John C. Allen, Jasmine Quan Lan Lew, Ngiap Chuan Tan, Tazeen H. Jafar

**Affiliations:** 1 Program in Health Services and Systems Research, Duke-NUS Medical School, 8 College Road, Singapore, Singapore; 2 Health Services Research Centre, SingHealth, Singapore, Singapore; 3 Center for Quantitative Medicine, Office of Clinical Sciences, Duke-NUS Medical School, Singapore, Singapore; 4 Heal Doctors, Los Angeles, CA USA; 5 SingHealth Polyclinics, Singapore, Singapore; 6 SingHealth-Duke NUS Family Academic Clinical Program, Singapore, Singapore; 7 Department of Renal Medicine, Singapore General Hospital, Singapore, Singapore; 8 Duke Global Health Institute, Duke University, Durham, NC USA

**Keywords:** Chronic kidney disease, End-stage kidney disease, Kidney failure risk equation, Prediction, Southeast Asia

## Abstract

**Background:**

Patients with chronic kidney disease (CKD) are at high risk of end-stage kidney disease (ESKD). The Kidney Failure Risk Equation (KFRE), which predicts ESKD risk among patients with CKD, has not been validated in primary care clinics in Southeast Asia (SEA). Therefore, we aimed to (1) evaluate the performance of existing KFRE equations, (2) recalibrate KFRE for better predictive precision, and (3) identify optimally feasible KFRE thresholds for nephrologist referral and dialysis planning in SEA.

**Methods:**

All patients with CKD visiting nine primary care clinics from 2010 to 2013 in Singapore were included and applied 4-variable KFRE equations incorporating age, sex, estimated glomerular filtration rate (eGFR), and albumin-to-creatinine ratio (ACR). ESKD onset within two and five years were acquired via linkage to the Singapore Renal Registry. A weighted Brier score (the squared difference between observed vs predicted ESKD risks), bias (the median difference between observed vs predicted ESKD risks) and precision (the interquartile range of the bias) were used to select the best-calibrated KFRE equation.

**Results:**

The recalibrated KFRE (named Recalibrated Pooled KFRE SEA) performed better than existing and other recalibrated KFRE equations in terms of having a smaller Brier score (square root: 2.8% vs. 4.0–9.3% at 5 years; 2.0% vs. 6.1–9.1% at 2 years), less bias (2.5% vs. 3.3–5.2% at 5 years; 1.8% vs. 3.2–3.6% at 2 years), and improved precision (0.5% vs. 1.7–5.2% at 5 years; 0.5% vs. 3.8–4.2% at 2 years). Area under ROC curve for the Recalibrated Pooled KFRE SEA equations were 0.94 (95% confidence interval [CI]: 0.93 to 0.95) at 5 years and 0.96 (95% CI: 0.95 to 0.97) at 2 years. The optimally feasible KFRE thresholds were > 10–16% for 5-year nephrologist referral and > 45% for 2-year dialysis planning. Using the Recalibrated Pooled KFRE SEA, an estimated 82 and 89% ESKD events were included among 10% of subjects at highest estimated risk of ESKD at 5-year and 2-year, respectively.

**Conclusions:**

The Recalibrated Pooled KFRE SEA performs better than existing KFREs and warrants implementation in primary care settings in SEA.

## Background

According to the Global Burden of Disease Study 2015, total mortality for chronic kidney disease (CKD) rose by 31.7% from 2005 to 2015 worldwide [1]. CKD stage 3 or worse (estimated glomerular filtration rate [eGFR] < 60 ml/min/1.73m^2^) is associated with increased risk of cardiovascular disease (CVD) and end-stage kidney disease (ESKD) that requires costly therapy including dialysis or kidney transplantation [2, 3].

Timely referral to nephrologists has shown to improve survival on dialysis [4] and reduce medical costs among patients who begin renal replacement therapy [5]. Although a variety of factors may influence a decision for nephrologist referral, typical eGFR thresholds in clinical guidelines have varied from < 30, < 45 to < 60 mL/min/1.73 m^2^ [6–8]. Based on experts’ opinions, a systematic review suggested that referral at eGFR < 60 ml/min/1.73m^2^ is likely to be more cost-effective than at eGFR < 40 ml/min/1.73m^2^ [9]. However, automated referrals for non-dialysis CKD have not been instituted in clinical practice and would likely overwhelm the health system as nephrologists are in short supply globally [10], with relative numbers of nephrologists ranging from 1 per million population in Southeast Asia to 31 per million in Western Europe [10]. Heavy nephrologist caseload has been associated with mortality of dialysis patients [11], and less timely access to treatment for patients at higher risk of ESKD [12–15]. Therefore, accurate prediction scores to identify high-risk patients for ESKD are vital for efficient patient triage, decreasing waiting time and allocating limited resources to patients at highest risk. In 2011, a predictive model called the Kidney Failure Risk Equation (KFRE) incorporating four variables (age, sex, eGFR, urine albumin-to-creatinine ratio [ACR]) or eight variables (age, sex, eGFR, ACR, serum calcium, phosphate, bicarbonate, and albumin) [16] was developed with excellent predictive performance for ESKD risk in a Canadian population [16]. Subsequently, the Original KFRE equation has been validated in more than 30 countries [17–22], recalibrated for non-North Americans using primarily European populations, and a Pooled KFRE equation has also been developed [17]. However, these KFRE equations were developed and evaluated primarily in patients visiting the nephrology clinics [16–18]. CKD is largely asympotmatic, and the vast majority of patients (up to 90%), especially with earlier stages of CKD, are unaware of their conditions [23]. Therefore, a well-performing KFRE would be highly relevant to the primary care settings to identify the fast progressors to ESKD. The Southeast Asian (SEA) population has been shown to have a heavy burden of ESKD [24] and may experience faster progression of CKD to ESKD compared to Caucasians [25–27]. However, the existing KFRE equations have not been evaluated in the SEA population.

Thus, we aimed to (1) compare performances of existing KFRE equations in a multi-ethnic population visiting primary care clinics in Singapore, (2) recalibrate KFRE to improve predictive precision for use in the SEA population, and (3) determine the optimally feasible KFRE thresholds to guide nephrologist referral and dialysis planning in SEA.

## Methods

### Study population

Singapore is a multi-ethnic country with major ethnic groups of Chinese, Malays and Indians. In 2017, there were 18 polyclinics (primary care clinics) located throughout Singapore, where about 60% Singaporeans with major risk factors for CKD (hypertension and diabetes) sought care [28]. The 18 polyclinics were managed by two major healthcare groups (the SingHealth and the National Healthcare groups) before 2017, and the current study was derived from the electronic health records (EHR) at 9 SingHealth polyclinics at the time of study with the follow-up durations of two and five years.

**Fig. 1:**
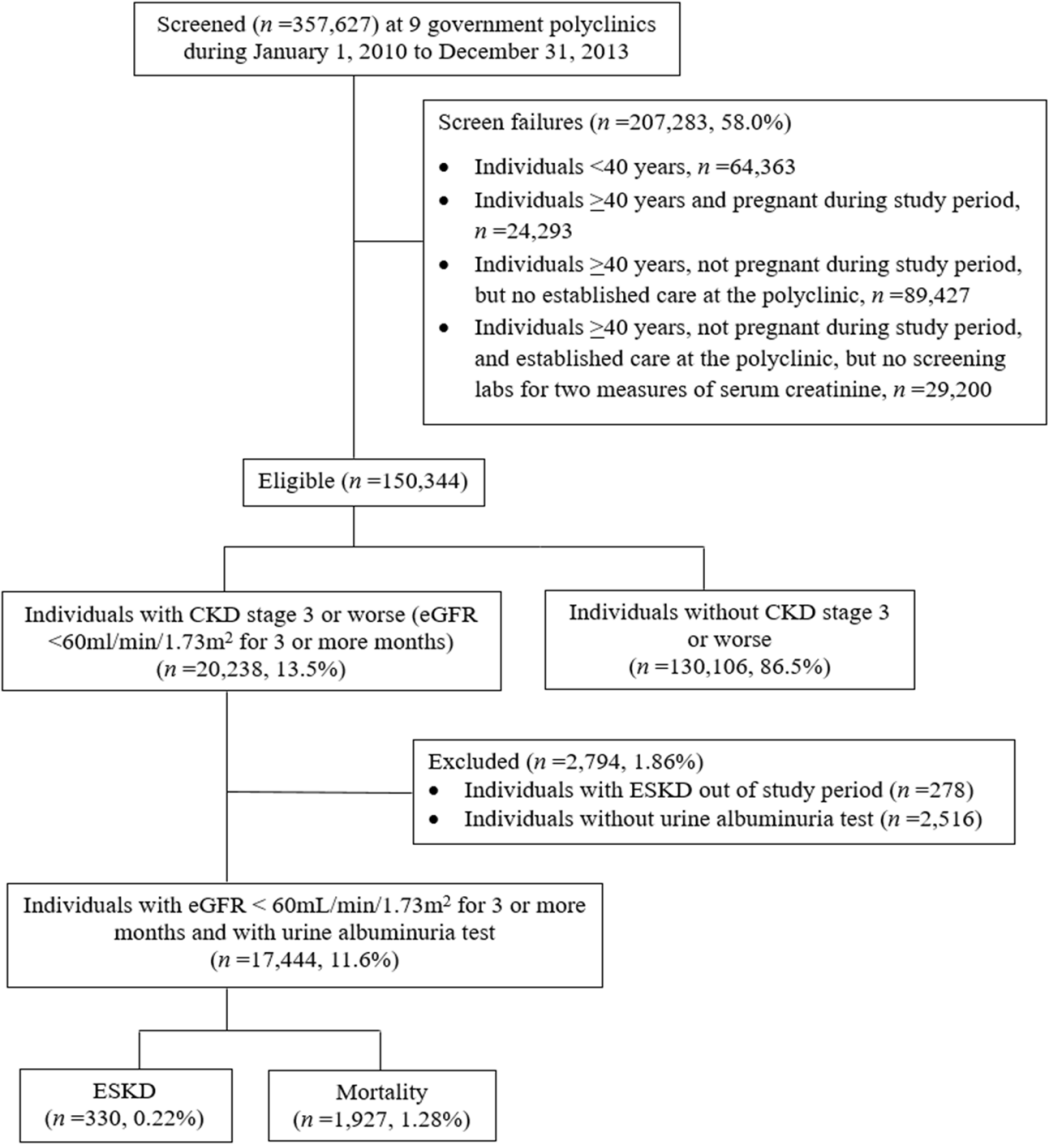
Flowchart of the 2-year follow-up study design. Abbreviations: CKD, chronic kidney disease; eGFR, estimated glomerular filtration rate; ESKD, end-stage kidney disease

**Fig. 2:**
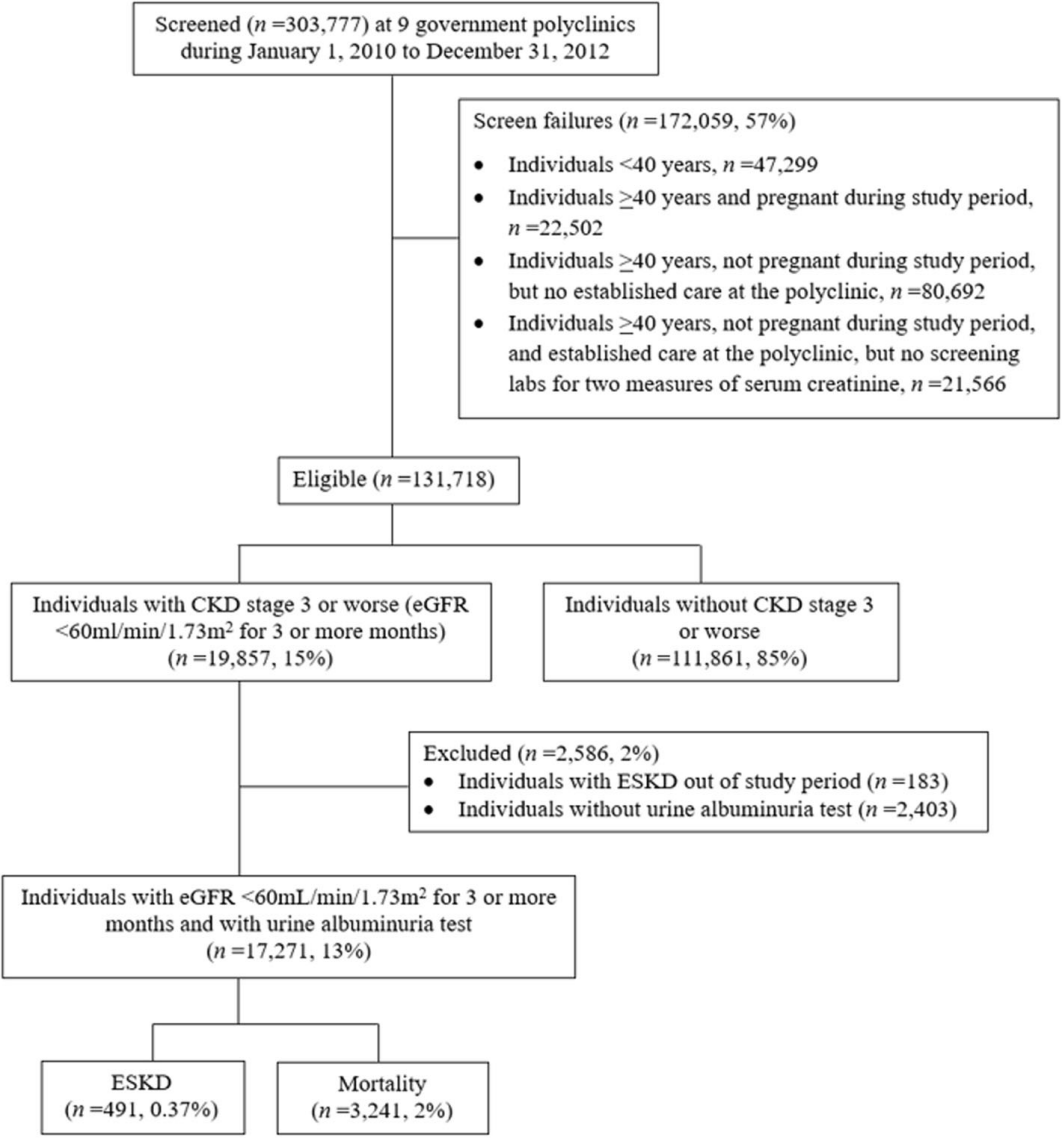
Flowchart of the 5-year follow-up study design. Abbreviations: CKD, chronic kidney disease; eGFR, estimated glomerular filtration rate; ESKD, end-stage kidney disease

For the 2-year follow-up, we included all 357,627 patients who visited the nine primary care clinics from January 1, 2010 to December 31, 2013 for eligibility screening. Eligibility criteria were 1) age ≥ 40 years, 2) not pregnant, 3) visited any primary care clinic at least twice with two visits at least 1 year apart, and 4) had ≥2 serum creatinine measurements taken at least 3 months apart to calculate eGFR by CKD-EPI equation [29] to screen for CKD. During the baseline screening window, if a patient met the eligibility criteria, he or she was immediately followed up on the date of recruitment. All other exposures including age, sex and urine albumin assessment were collected at the same time. During the follow-up, a patient may have died, or develop ESKD within the follow-up period, or remain ESKD-free at the end of the follow-up. Among the 150,344 eligible patients, 20,238 (14%) had persistent reductions in eGFR of < 60 mL/min/1.73m^2^. For the 5-year follow-up, we screened all 303,777 patients who visited the nine primary care clinics from January 1, 2010 to December 31, 2012, and 131,718 patients met the screening criteria. Among them, 19,857 (15%) had eGFR < 60 mL/min/1.73m^2^. Noteworthy, compared to the 5-year follow up, the 2-year follow-up had a shorter duration; thus, we extended the screening period to 3 years to capture more patients with ESKD. The flowcharts detailing the study design are shown in Fig. 1 and Fig. 2. The SingHealth Centralized Institutional Review Board granted ethics approval and consent waiver.

### Outcomes assessment

Incident ESKD cases and deaths were determined via linkage with the population-based Singapore Renal Registry and the Singapore Registry of Births and Deaths. Linkage was accomplished by matching the National Registration Identity Card number assigned to each citizen or permanent resident in Singapore and then verified by name. The Singapore Renal Registry identified ESKD as meeting one of the following criteria: 1) serum creatinine ≥880 μmol/L (10 mg/dL), 2) eGFR (based on either the Modification of Diet in Renal Disease Study equation [30], the Cockcroft Gault equation [30], or 24-h creatinine clearance) < 15 mL/min/1.73 m^2^, 3) undergoing peritoneal dialysis or haemodialysis, or 4) kidney transplantation has been undertaken [31]. A diagnosis of ESKD required any of the criteria 1) - 3) above to be persistent for at least 3 months [6, 31].

### Exposures assessment and KFRE validation

Information on age, gender, ethnicity, lifestyle factors, and co-morbidities was obtained from EHR data. Patients with hypertension or diabetes received blood and urine laboratory tests annually at primary care clinics. Creatinine measurements were calibrated to be traceable to isotope dilution mass spectrometry (IDMS) standardization [32]. Urine albumin was measured as dipstick [33] in all patients and expressed as log-transformed ACR to be used in the KFRE (negative as 9, trace as 43, “+” as 81, “++” as 315, “>++” as 1073) [17, 34, 35].

We excluded patients without ACR (5-year, *n* = 2403; 2-year, *n* = 2516) and those developed ESKD before study baseline (5-year, *n* = 183; 2-year, *n* = 278), leaving 17,271 and 17,444 patients in the 5-year and 2-year cohorts for analysis. No missing data existed for other exposure variables (eGFR, age and sex). In calculating ESKD risks, we applied the existing 4-variable KFRE equations developed by Tangri et al. [17] (Additional file 1 and Additional file 2) based on the first eGFR and urine dipstick measurements. Three KFREs (Original KFRE, Original KFRE Calibrated for North American, Original KFRE Calibrated for non-north American) had the same regression coefficients but different baseline hazards, and the Pooled KFRE equation had both different regression coefficients and baseline hazards from the other three KFRE equations [17] (Additional file 1 and Additional file 2).

### Statistical analysis

#### Recalibration of KFRE equations for SEA

We fit a Cox proportional hazards model using the same variables included in the Original KFRE (age, 70 years; 56% men, eGFR, 36 mL/min/1.73 m^2^, ACR 170 mg/g) [16, 17] and explored the baseline hazard and regression coefficients for the recalibration of KFRE equations for SEA population. We formed one recalibrated KFRE equation (Recalibrated Original KFRE SEA 1) by changing the baseline hazard in the Original KFRE, and formed one recalibrated KFRE (Recalibrated Original KFRE SEA 2) by changing both the baseline hazard and regression coefficients in the Original KFRE (Additional file 1 and Additional file 2). We also recalibrated the Pooled KFRE by changing the baseline hazard in a stepwise manner to look for an equation with the best calibration (Recalibrated Pooled KFRE SEA) (Additional file 1, Additional file 2 and Additional file 3).

#### Metrics for equation performance

The metrics used to compare the calibration (how closely the predicted risks agree with the observed risks) among different KFRE equations were the Brier score [36], bias [37], and precision [37]. The Brier score was calculated as the squared difference of mean observed minus predicted risks [36] weighted according to the sample sizes in five risk categories (for 5 years, 0 to < 5%, 5 to < 15%, 15 to < 25%, 25 to < 50%, and ≥ 50%; for 2 years, 0 to < 2%, 2 to < 6%, 6 to < 10%, 10 to < 20%, and ≥ 20%) adopted from prior studies [16, 17]. Bias was expressed as the median difference between observed minus predicted risks [37] and precision was the interquartile range of bias [37]. A KFRE equation with the lowest score of all three metrics would be chosen as the best-calibrated equation.

#### Discrimination and risk reclassification

We used area under receiver operating characteristic curve (AUC) of the best-calibrated KFRE equation as a continuous variable to that of continuous eGFR. We also used category-free net reclassification improvement (NRI) to compare the KFRE with eGFR: we assessed that how many more patients with ESKD were correctly assigned to higher predicted risks, as well as patients without ESKD to lower risks by using KFRE versus eGFR [38]. We also applied AUC and NRI to compare between different KFRE equations.

#### Proportion of cases followed [PCF(*p*)] and proportion of the population needed to be follow [PNF(*q*)]

PCF(*p*) and PNF(*q*) were two recently developed measures that are highly relevant to the decision making in public health. PCF(*p*) represents the proportion of individuals who will develop disease who are included in the proportion *p* of individuals in the population of the highest risk, and PNF(*q*) is the proportion of the general population at highest risk that one needs to follow in order that a proportion *q* of those destined to become cases will be followed [39, 40]. For the equation with best calibration and predictive performance, we further calculated the PCF(*p*) and PNF(*q*) for the public health implication.

#### Explore the optimally feasible KFRE threshold in southeast Asians

For the best-calibrated KFRE equation, we applied the Youden Index to determine a statistically dichotomous risk threshold (‘low’ versus ‘high’) [41]. The statistical threshold had the highest summation of the sensitivity and specificity. We compared the statistical KFRE thresholds identified in the current population to the 3, 5, and 10% at 5 years [17, 22] and 20 and 40% at 2 years [17], and also compared KFRE-based criteria to eGFR 30–60 ml/min/1.73m^2^ at 5 years and 20 ml/min/1.73m^2^ at 2 years suggested by Tangri et al. [42]. We also compared KFRE thresholds with eGFRs that captured the same proportions of patients in this population to test the robustness of the results. The selection of the optimally feasible thresholds was based on sensitivity [43], specificity [43], positive predictive value (PPV) [43], negative predictive value (NPV) [43], positive likelihood ratio (LR+) [44], negative likelihood ratio (LR-) [44], and balanced by service-related considerations of (i) total number of patients with CKD referred to nephrologists, and (ii) number of patients with CKD needed to be evaluated by nephrologists to identify one patient who will progress to ESKD.

#### Stratified and sensitivity analyses

We conducted stratified analyses by age (40–75 vs. > 75 years), gender, ethnicity, type 2 diabetes mellitus status, and CKD stages (stage 3 and 4) for both 5-year and 2-year ESKD risks. To be consistent with Tangri et al., we also defined ESKD by limiting to those who started dialysis and received kidney transplantation only, and calculated the AUC of the best-calibrated KFRE equation. Since some patients died before the onset of ESKD of other causes, we evaluated the impact of competing risks of death on the KFRE risk prediction using the proportional hazards model proposed by Fine and Gray [45] compared to the non-competing risk model using the conventional Cox regression model. We used STATA software version 13.0 (Stata Corp, College Station, Texas) for all data analyses. Statistical significance was set at *P* ≤ 0.05.

## Results

### Baseline characteristics

Baseline characteristics of patients at 5- and 2-year ESKD risks are shown in Table 1 and Additional file 4. In both cohorts, the mean age was about 75 years, and approximately 50% were men, 80% were Chinese, 4% were Indians and 13% were Malays. At 5 years, 491 (2.8%) patients developed ESKD, among whom 147 (0.9%) with stage 3 CKD progressed to ESKD with a median time of 2.42 (range: 0.04–3.99) years, and 261 (1.5%) with stage 4 CKD developed ESKD in 1.78 (range: 0.09–3.93) years (Table 1). At 2 years, 330 (1.9%) developed ESKD; the median progression time from CKD to ESKD was 2.00 (range: 0.07–2.00) years among 48 (0.3%) patients with stage 3 CKD, and it was 1.13 (range: 0.01–2.00) years among 188 (1.1%) patients with stage 4 CKD (Additional file 4). In both cohorts, compared with non-ESKD patients, those with ESKD tended to be younger and have higher BMI, and were more likely to have type 2 diabetes mellitus, live in government housing and have a history of smoking (Table 1 and Additional file 4). For the 5-year cohort, compared to patients excluded for lack of established care or missing a second measurement of serum creatinine (*n* = 102,258), those included in the analysis (*n* = 131,718) were more likely to have hypertension (87.5% vs 32.5%, *P* < 0.001) or type 2 diabetes mellitus (44.6% vs 14.2%, *P* < 0.001).

**Table 1:** Baseline characteristics of patients with chronic kidney disease stage 3–5 with 5-year follow-up^a^

	Total patients with CKD (*n* = 17,271)	Patients with ESKD (*n* = 491)	Patients without ESKD (*n* = 16,780)
CKD stages, *n* (%)			
Stage 3 CKD (30 ≤ eGFR< 60 mL/min/1.73m^2^)	15,313 (89)	147 (30)	15,166 (90)
Stage 4 CKD (15 ≤ eGFR< 30 mL/min/1.73m^2^)	1833 (11)	261 (53)	1572 (9)
Stage 5 CKD (eGFR< 15 mL/min/1.73m^2^)	125 (0.72)	83 (17)	42 (0.25)
Demographics			
Age (years), mean (SD)	75 (9)	70 (10)	75 (9)
Gender, *n* (%)			
Men	8461 (49)	233 (48)	8228 (49)
Women	8810 (51)	258 (53)	8552 (51)
Ethnicity, *n* (%)			
Chinese	13,837 (80)	356 (73)	13,481 (80)
Indians	708 (4)	22 (4)	686 (4)
Malays	2155 (13)	90 (18)	2065 (12)
Others	571 (3)	23 (5)	548 (4)
Lifestyle factors			
Government housing, *n* (%)	15,348 (89)	456 (93)	14,892 (89)
Past or current smoker, *n* (%)	1097 (6)	42 (9)	1055 (6)
BMI (kg/m^2^), mean (SD)	25.5 (4.52)	26.3 (4.31)	25.5 (4.52)
Known co-morbidities^b^			
Physician-diagnosed diabetes mellitus, *n* (%)	10,129 (59)	396 (81)	9733 (58)
Physician-diagnosed hypertension, *n* (%)	17,019 (99)	488 (99)	16,531 (99)
Physician-diagnosed cardiovascular disease, *n* (%)	4354 (25)	140 (29)	4214 (25)
Physician-diagnosed stroke, *n* (%)	2118 (12)	62 (13)	2056 (12)
Recalibrated Pooled KFRE SEA equation^c^			
> 3%, *n* (%)	8229 (48)	482 (98)	7817 (47)
> 5%, *n* (%)	6017 (35)	475 (97)	5542 (33)
> 10%, *n* (%)	3506 (20)	448 (91)	3058 (18)

^a^Data are expressed as mean (standard deviation) for continuous variables and *n* (percentage) for categorical variables. CKD was defined as CKD-EPI eGFR < 60 mL/min/1.73m^2^

^b^Known co-morbidities as documented by physicians in the electronic health record

^c^The Recalibrated Pooled KFRE SEA equation at 5-year ESKD risk was calculated as: 1–0.8362 ^ exp. (− 0.2245 × (age/10–7.036) + 0.3212 × (male - 0.5642) - 0.4553 × (eGFR/5–7.222) + 0.4469 × (lnACR - 5.137))

Abbreviation: *ACR* albumin-to-creatinine ratio, *BMI* body mass index, *CKD* chronic kidney disease, *CKD-EPI* Chronic Kidney Disease Epidemiology Collaboration, *eGFR* estimated glomerular filtration rate, *ESKD* end-stage kidney disease, *KFRE* Kidney Failure Risk Equation, *SEA* Southeast Asia, *SD* standard deviation

### Recalibrated KFRE equations

The recalibrated KFRE equations for 5- and 2-year risks are listed in Additional file 1 and Additional file 2. The baseline hazard from the Cox proportional hazard model for the current population was 0.9595 for 5-year and 0.9822 for 2-year risks (used for Recalibrated Original KFRE SEA 1). The regression coefficients for age, gender, and ACR are listed in Additional file 1 and Additional file 2, respectively (used for Recalibrated Original KFRE SEA 2). For the recalibrated Pooled KFRE equation, the best-calibrated baseline hazard was 0.8362 at 5-year and 0.8976 at 2-year risks (used for Recalibrated Pooled KFRE SEA) (Additional file 1, Additional file 2 and Additional file 3).

### Comparison of performance

The Recalibrated Pooled KFRE SEA had the best calibration among all KFREs in terms of having the smallest Brier score (squared difference of mean observed minus predicted risks [36]; square root: 2.8% vs. 4.0–9.3% at 5 years; 2.0% vs. 6.1–9.1% at 2 years), the least bias (median difference between observed minus predicted risks [37]; 2.5% [− 2.0–4.5%] vs. 3.3–5.2% at 5 years; 1.8% [− 1.7–3.5%] vs. 3.2–3.6% at 2 years), and the best precision (interquartile range of bias [37]; 0.5% vs.1.7–5.2% at 5 years; 0.5% vs. 3.5–4.2% at 2 years) (Table 2). Compared to the observed risks, the Recalibrated Pooled KFRE SEA slightly underestimated the predicted ESKD risks at lower KFRE risk categories (< 25% at 5 years; < 20% at 2 years), and slightly overestimated the predicted risks at higher risk categories (≥25% at 5 years; ≥20% at 2 years) (Additional file 5).

**Table 2:** Comparison of calibration performances of existing KFRE equations and recalibrated KFRE equations at 5-year and 2-year risks of end-stage kidney disease

	KFRE equations	Brier score^a^ (square root)	Bias^a^ (95% CI)	Precision^a^
5-year risk				
Existing KFRE equations	Original KFRE [17]	6.2%	4.5% (− 1.4–5.9%)	4.3%
	Original KFRE calibrated for north American [17]	4.8%	4.1% (0.2–5.8%)	2.6%
	Original KFRE calibrated for non-north American [17]	7.2%	4.8% (− 2.8–7.6%)	4.3%
	Pooled KFRE [17]	4.0%	3.3% (− 0.1–6.7%)	1.7%
Recalibrated KFRE equations	Recalibrated Original KFRE SEA 1^b^	9.3%	5.1% (− 6.9–12.0%)	5.1%
	Recalibrated Original KFRE SEA 2^c^	7.9%	5.2% (−4.4–9.6%)	5.2%
	Recalibrated Pooled KFRE SEA^d^	2.8%	2.5% (−2.0–4.5%)	0.5%
2-year risk				
Existing KFRE equations	Original KFRE [17]	7.9%	3.4% (−7.8–11.2%)	3.8%
	Original KFRE calibrated for north American [17]	7.9%	3.4% (− 7.8–11.2%)	3.8%
	Original KFRE calibrated for non-north American [17]	9.1%	3.5% (−10.4%-13.95)	4.2%
	Pooled KFRE [17]	7.9%	3.2% (−8.7–11.9%)	3.5%
Recalibrated KFRE equations	Recalibrated Original KFRE SEA 1^e^	9.0%	3.5% (−10.0–13.5%)	4.1%
	Recalibrated Original KFRE SEA 2^f^	6.1%	3.6% (−5.1–8.7%)	3.9%
	Recalibrated Pooled KFRE SEA^g^	2.0%	1.8% (−1.7–3.5%)	0.5%

^a^Brier score is calculated as the squared difference of mean observed minus predicted risks. Bias is calculated as the observed minus predicted risks. Precision is the interquartile range of the bias. The KFRE equation with a lower score of all three metrics is the best-calibrated equation

^b^The Recalibrated Original KFRE SEA 1 at 5-year ESKD risk was calculated as: 1–0.9595 ^ exp. (− 0.2201 × (age/10–7.036) + 0.2467 × (male – 0.5642) – 0.5567 × (eGFR/5–7.222) + 0.4510 × (logACR – 5.137))

^c^The Recalibrated Original KFRE SEA 2 at 5-year ESKD risk was calculated as: 1–0.9595 ^ exp. (− 0.4734 × (age/10–7.036) + 0.0119 × (male – 0.5642) – 0.6990 × (eGFR/5–7.222) + 0.6159 × (logACR – 5.137))

^d^The Recalibrated Pooled KFRE SEA at 5-year ESKD risk was calculated as: 1–0.8362 ^ exp. (− 0.2245 × (age/10–7.036) + 0.3212 × (male – 0.5642) – 0.4553 × (eGFR/5–7.222) + 0.4469 × (logACR – 5.137))

^e^The Recalibrated Original KFRE SEA 1 at 2-year ESKD risk was calculated as: 1–0.9822 ^ exp. (− 0.2201 × (age/10–7.036) + 0.2467 × (male – 0.5642) – 0.5567 × (eGFR/5–7.222) + 0.4510 × (logACR – 5.137))

^f^The Recalibrated Original KFRE SEA 2 at 2-year ESKD risk was calculated as: 1–0.9822 ^ exp. (− 0.4416 × (age/10–7.036) – 0.0723 × (male – 0.5642) – 0.8232 × (eGFR/5–7.222) + 0.5418 × (logACR – 5.137))

^g^The Recalibrated Pooled KFRE SEA at 2-year ESKD risk was calculated as: 1–0.8976 ^ exp. (− 0.2245 × (age/10–7.036) + 0.3212 × (male – 0.5642) – 0.4553 × (eGFR/5–7.222) + 0.4469 × (logACR – 5.137))

Abbreviations: *CI* confidence interval, *ESKD* end-stage kidney disease, *KFRE* Kidney Failure Risk Equation, *SEA* Southeast Asia

### Discrimination, thresholds and NRI

The AUCs and 95% CIs for the Recalibrated Pooled KFRE SEA were 0.94 (0.93 to 0.95) at 5-year ESKD risks and 0.96 (0.95 to 0.97) at 2-year, which were statistically significantly higher than eGFR alone (0.89 [0.88 to 0.91] at 5 years; 0.93 [0.92 to 0.95] at 2 years) (Tables 3,4 and 5 & Additional file 6). In addition, the AUCs for other KFRE equations were the same with the Recalibrated Pooled KFRE SEA (Table 3). Consistent with the AUC results, the NRIs comparing Recalibrated Pooled KFRE SEA to other KFRE equations showed similar performances (NRI ranging from − 0.23-1.72%) (Table 3).

**Table 3:** Comparison of predictive performances of existing KFRE equations and recalibrated KFRE equations at 5-year and 2-year risks of ESKD

	KFRE equations	AUC (95% CI)	NRI^a^ (95% CI)
5-year risk			
Existing KFRE equations	Original KFRE [17]	0.94 (0.93–0.95)	0.19% (0.12–0.30%)
	Original KFRE calibrated for north American [17]	0.94 (0.93–0.95)	0.19% (0.12–0.30%)
	Original KFRE calibrated for non-north American [17]	0.94 (0.93–0.95)	0.21% (0.12–0.30%)
	Pooled KFRE [17]	0.94 (0.93–0.95)	−0.11% (− 0.20%--0.06%)
Recalibrated KFRE equations	Recalibrated Original KFRE SEA 1^b^	0.94 (0.93–0.95)	0.21% (0.14–0.32%)
	Recalibrated Original KFRE SEA 2^c^	0.94 (0.93–0.95)	1.72% (1.48–1.99%)
	Recalibrated Pooled KFRE SEA^d^	0.94 (0.93–0.95)	–
2-year risk			
Existing KFRE equations	Original KFRE [17]	0.96 (0.95–0.97)	0.03% (0.01–0.09%)
	Original KFRE calibrated for north American [17]	0.96 (0.95–0.97)	0.01% (0–0.06%)
	Original KFRE calibrated for non-north American [17]	0.96 (0.95–0.97)	0.01% (0–0.06%)
	Pooled KFRE [17]	0.96 (0.95–0.97)	0.01% (0–0.06%)
Recalibrated KFRE equations	Recalibrated Original KFRE SEA 1^e^	0.96 (0.95–0.97)	0.31% (0.22–0.44%)
	Recalibrated Original KFRE SEA 2^f^	0.96 (0.95–0.97)	−0.23% (− 0.35%--0.15%)
	Recalibrated Pooled KFRE SEA^g^	0.96 (0.95–0.97)	–

^a^NRI was compared between Recalibrated Pooled KFRE SEA over all other KFRE equations using individual statistical threshold identified by Youden Index to dichotomize

^b^The Recalibrated Original KFRE SEA 1 at 5-year ESKD risk was calculated as: 1–0.9595 ^ exp. (− 0.2201 × (age/10–7.036) + 0.2467 × (male – 0.5642) – 0.5567 × (eGFR/5–7.222) + 0.4510 × (logACR – 5.137))

^c^The Recalibrated Original KFRE SEA 2 at 5-year ESKD risk was calculated as: 1–0.9595 ^ exp. (− 0.4734 × (age/10–7.036) + 0.0119 × (male – 0.5642) – 0.6990 × (eGFR/5–7.222) + 0.6159 × (logACR – 5.137))

^d^The Recalibrated Pooled KFRE SEA at 5-year ESKD risk was calculated as: 1–0.8362 ^ exp. (− 0.2245 × (age/10–7.036) + 0.3212 × (male – 0.5642) – 0.4553 × (eGFR/5–7.222) + 0.4469 × (logACR – 5.137))

^e^The Recalibrated Original KFRE SEA 1 at 2-year ESKD risk was calculated as: 1–0.9822 ^ exp. (− 0.2201 × (age/10–7.036) + 0.2467 × (male – 0.5642) – 0.5567 × (eGFR/5–7.222) + 0.4510 × (logACR – 5.137))

^f^The Recalibrated Original KFRE SEA 2 at 2-year ESKD risk was calculated as: 1–0.9822 ^ exp. (− 0.4416 × (age/10–7.036) – 0.0723 × (male – 0.5642) – 0.8232 × (eGFR/5–7.222) + 0.5418 × (logACR – 5.137))

^g^The Recalibrated Pooled KFRE SEA at 2-year ESKD risk was calculated as: 1–0.8976 ^ exp. (− 0.2245 × (age/10–7.036) + 0.3212 × (male – 0.5642) – 0.4553 × (eGFR/5–7.222) + 0.4469 × (logACR – 5.137))

Abbreviations: *AUC* area under the receiver operating characteristic curve, *CI* confidence interval, *ESKD* end-stage kidney disease, *KFRE* Kidney Failure Risk Equation, *NRI* net reclassification improvement, *SEA* Southeast Asia

**Table 4:** Summary statistics for selected thresholds of Recalibrated Pooled KFRE SEA equation for 5-year risk of end-stage kidney disease

Threshold	Recalibrated Pooled KFRE SEA equation^a^ AUC (95% CI): 0.94 (0.93–0.95)				CKD-EPI eGFR mL/min/1.73m^2^ AUC (95% CI): 0.89 (0.88–0.91)				
	> 3%	> 5%	> 10%	> 16%	< 30	< 35.4	< 40	< 45	< 60
Cumulative *n* of patients	8299 (48%)	6017 (35%)	3506 (20%)	2308 (13%)	1958 (11%)	3506 (20%)	5283 (30%)	7813 (45%)	17,271 (100%)
*N* of patients with CKD needed for evaluation by nephrologists to find one ESKD case	19.7 (18.2–21.3)	12.7 (11.6–13.9)	7.8 (7.2–8.5)	5.6 (5.0–6.0)	5.7 (5.2–6.3)	8.7 (7.9–9.5)	12.2 (11.1–13.3)	16.9 (15.6–18.5)	35.2 (32.3–38.5)
Risk of ESKD	5.8% (5.3–6.3%)	7.9% (7.2–8.6%)	12.8% (11.7–13.9%)	18.0% (16.8–20.0%)	17.6% (16.0–19.3%)	11.5% (10.5–12.6%)	8.2% (7.5–9.0%)	5.9% (5.4–6.4%)	2.8% (2.6–3.1%)
Sensitivity (95% CI)	0.98 (0.97–0.99)	0.97 (0.96–0.98)	0.91 (0.90–0.92)	0.86 (0.85–0.87)	0.70 (0.68–0.72)	0.82 (0.80–0.84)	0.88 (0.87–0.89)	0.94 (0.93–0.95)	1.00 (0.99–1.01)
Specificity (95% CI)	0.53 (0.51–0.55)	0.67 (0.66–0.68)	0.82 (0.81–0.83)	0.89 (0.88–0.90)	0.90 (0.89–0.91)	0.82 (0.81–0.83)	0.71 (0.70–0.72)	0.56 (0.55–0.57)	0.01 (0.00–0.02)
PPV (95% CI)	0.05 (0.04–0.06)	0.07 (0.06–0.08)	0.10 (0.09–0.11)	0.16 (0.14–0.18)	0.18 (0.16–0.20)	0.11 (0.10–0.12)	0.08 (0.07–0.09)	0.06 (0.05–0.07)	0.03 (0.02–0.04)
NPV (95% CI)	0.999 (0.998–1.000)	0.998 (0.997–0.999)	0.997 (0.990–1.004)	0.995 (0.980–1.010)	0.990 (0.970–1.010)	0.994 (0.984–1.004)	0.995 (0.986–1.004)	0.997 (0.992–1.002)	1.000 (0.085–1.015)
LR+ (95% CI)	2.11 (2.08–2.17)	2.92 (2.86–3.03)	5.01 (5.00–5.26)	7.67 (7.14–8.33)	7.00 (6.67–7.69)	4.56 (4.46–4.66)	3.03 (2.94–3.13)	2.14 (2.08–2.17)	1.00 (0.99–1.01)
LR- (95% CI)	0.03 (0.02–0.04)	0.05 (0.04–0.06)	0.10 (0.09–0.11)	0.16 (0.14–0.18)	0.33 (0.31–0.35)	0.22 (0.21–0.23)	0.17 (0.16–0.18)	0.11 (0.10–0.12)	0.00 (− 0.01–0.01)

^a^The Recalibrated Pooled KFRE SEA equation for 5-year ESKD risk was calculated as: 1–0.8362 ^ exp. (− 0.2245 × (age/10–7.036) + 0.3212 × (male - 0.5642) - 0.4553 × (eGFR/5–7.222) + 0.4469 × (lnACR - 5.137)). The statistical threshold identified by Youden Index was 16%

Abbreviation: *ACR* albumin-to-creatinine ratio, *AUC* area under the receiver operating characteristic curve, *CI* confidence interval, *CKD-EPI* Chronic Kidney Disease Epidemiology Collaboration, *eGFR* estimated glomerular filtration rate, *ESKD* end-stage kidney disease, *KFRE* Kidney Failure Risk Equation, *LR+* positive likelihood ratio, *LR*- negative likelihood ratio, *NPV* negative predictive value, *PPV* positive predictive value, *SEA* Southeast Asia

**Table 5:** Summary statistics for selected thresholds of Recalibrated Pooled KFRE SEA equation for 2-year risk of end-stage kidney disease

Threshold	Recalibrated Pooled KFRE SEA equation^a^ AUC (95% CI): 0.96 (0.95–0.97)				CKD-EPI eGFR mL/min/1.73m^2^ AUC (95% CI): 0.93 (0.92–0.95)	
	> 9%	> 20%	> 40%	> 45%	< 18.8	< 20
Cumulative *n* of patients	2683 (15.4%)	1240 (7.1%)	531 (3.0%)	430 (2.5%)	430 (2.5%)	546 (3.1%)
*N* of patients with CKD needed for evaluation by nephrologists to find one ESKD case	8.8 (7.9–9.7)	4.6 (4.2–5.2)	2.7 (2.4–3.1)	2.4 (2.2–2.7)	2.8 (2.4–3.1)	3.2 (2.8–3.6)
Risk of ESKD	11.4% (10.3–12.7%)	21.6% (19.4–24.0%)	36.7% (32.7–40.9%)	41.6% (37.1–46.3%)	36.3% (31.9–40.9%)	31.3% (27.6–35.3%)
Sensitivity (95% CI)	0.93 (0.92–0.94)	0.81 (0.79–0.83)	0.59 (0.54–0.63)	0.54 (0.49–0.59)	0.47 (0.43–0.51)	0.52 (0.48–0.56)
Specificity (95% CI)	0.86 (0.85–0.87)	0.94 (0.93–0.95)	0.98 (0.97–0.99)	0.99 (0.98–1.00)	0.98 (0.97–0.99)	0.98 (0.97–0.99)
PPV (95% CI)	0.07 (0.06–0.08)	0.20 (0.19–0.21)	0.38 (0.37–0.39)	0.42 (0.37–0.47)	0.36 (0.32–0.44)	0.31 (0.27–0.35)
NPV (95% CI)	0.999 (0.990–1.008)	0.997 (0.987–1.007)	0.992 (0.985–0.999)	0.992 (0.982–1.002)	0.989 (0.979–0.999)	0.991 (0.989–1.001)
LR+ (95% CI)	6.68 (6.25–7.14)	14.3 (12.5–16.7)	30.1 (27.0–37.8)	36.8 (25.0–50.0)	23.5 (16.0–30.3)	26.0 (20.0–33.3)
LR- (95% CI)	0.08 (0.07–0.09)	0.20 (0.16–0.20)	0.41 (0.36–0.46)	0.47 (0.42–0.52)	0.54 (0.44–0.65)	0.49 (0.45–0.53)

^a^The Recalibrated Pooled KFRE SEA equation for 2-year ESKD risk was calculated as: 1–0.8976 ^ exp. (− 0.2245 × (age/10–7.036) + 0.3212 × (male - 0.5642) - 0.4553 × (eGFR/5–7.222) + 0.4469 × (lnACR - 5.137)). The statistical threshold identified by Youden Index was 9%

Abbreviation: *ACR* albumin-to-creatinine ratio, *AUC* area under the receiver operating characteristic curve, *CI* confidence interval, *CKD-EPI* Chronic Kidney Disease Epidemiology Collaboration, *eGFR* estimated glomerular filtration rate, *ESKD* end-stage kidney disease, *KFRE* Kidney Failure Risk Equation, *LR+* positive likelihood ratio, *LR-* negative likelihood ratio, *NPV* negative predictive value, *PPV* positive predictive value, *SEA* Southeast Asia

At 5 years, we used eGFR 40 mL/min/1.73m^2^ (sensitivity 0.88, specificity 0.71) as reference to compare with thresholds of Recalibrated Pooled KFRE SEA because the eGFR 30 mL/min/1.73m^2^ sensitivity was suboptimal (0.70 [0.68–0.72]), and eGFR 45 and 60 mL/min/1.73m^2^ specificities were low (0.56 [0.55–0.57] and 0.01 [0.00–0.02]). Using eGFR < 40 mL/min/1.73m^2^ would identify 5283 (30%) patients requiring referral to a nephrologist, and this number was substantially higher than the 3506 (20%) of KFRE > 10%, and 2308 (13%) of the Youden Index-determined KFRE > 16%. Moreover, using KFRE thresholds ranging 10–16%, nephrologists need to evaluate 5.6 (5.0–6.0) to 7.8 (7.2–8.5) patients with CKD to find one ESKD case, and this resulted in higher referral efficiency than the 12.2 (11.1–13.3) patients using eGFR 40 mL/min/1.73m^2^. In addition to the higher referral efficiency, KFRE thresholds 10–16% also had similar sensitivity (0.86 [0.85–0.87] to 0.91 [0.90–0.92] vs. 0.88 [0.87–0.89]), higher specificity (0.82 [0.81–0.83] to 0.89 [0.88–0.90] vs. 0.71 [0.70–0.72]), higher PPV (0.10 [0.09–0.11] to 0.16 [0.14–0.18] vs. 0.08 [0.07–0.09]), similar NPV (0.995 [0.980–1.010] to 0.997 [0.990–1.004] vs. 0.995 [0.986–1.004]), and higher LR+ (5.01 [5.00–5.26] to 7.67 [7.14–8.33] vs. 3.03 [2.94–3.13]) compared to eGFR 40 mL/min/1.73m^2^ (Table 4).

In the 2-year cohort, using threshold of Recalibrated Pooled KFRE SEA > 45% had higher clinical efficiency than eGFR < 20 mL/min/1.73m^2^ in terms of fewer referral number to nephrologist (430 vs. 546), and similar number of patients to be evaluated by nephrologists to find one ESKD case (2.7 [2.4–3.1] vs. 3.2 [2.8–3.6]). In addition, KFRE threshold 45% also had similar sensitivity (0.54 [0.49–0.59] vs. 0.52 [0.48–0.56]), similar specificty (0.99 [0.98–1.00] vs. 0.98 [0.97–0.99]), similar NPV (0.992 [0.982–1.002] vs. 0.991 [0.989–1.001]), similar LR+ (36.8 [25.0–50.0] vs. 26.0 [20.0–33.3]), and higher PPV (0.42 [0.37–0.47] vs. 0.31 [0.27–0.35]) compared to those of eGFR 20 mL/min/1.73m^2^ (Table 5), suggesting a marginal superiority.

The Recalibrated Pooled KFRE SEA resulted in statistically significant improvement in NRI over eGFR alone in predicting ESKD. At 5 years, NRIs were ≥ 12.6% (10.6–15.3%) for KFRE 10–16% compared to eGFR 30, 40 and 45 mL/min/1.73m^2^ (Additional file 7). At 2 years, the NRI was 3.14% (2.86, 3.43%) for KFRE 45% compared to eGFR 20 mL/min/1.73m^2^ (Additional file 8).

In addition to the traditional eGFR cut-off values, we also compared the abovementioned KFRE thresholds to the eGFR cut-offs that captured the same proportions of patients in this population. Thus, at 5 years, the KFRE > 10% corresponded to eGFR < 35.4 mL/min/1.73m^2^, and the KFRE > 16% corresponded to eGFR < 30 mL/min/1.73m^2^ approximately (Table 4); at 2 years, the KFRE> 45% corresponded to eGFR< 18.8 mL/min/1.73m^2^ (Table 5). As a result, at 5 years, KFRE 10 and 16% had higher sensitivity and lower negative likelihood ratios compared to respective eGFR cut-off values, while other statistics (specificity, PPV, NPV, positive likelihood ratio and referral efficiency) remained the same (Table 4). At 2 years, KFRE > 45% had similar sensitivity, specificity, PPV, NPV, positive likelihood ratio, negative likelihood ratio, and referral efficiency compared to eGFR < 18.8 mL/min/1.73m^2^ (Table 5). However, all of the KFRE thresholds resulted in a positive NRI compared to the corresponding eGFR cut-off points (≥7.06% [6.77–7.34%]) (Additional file 7 and Additional file 8), indicating the robustness of the superiority of KFRE in clinical utility.

**Fig. 3:**
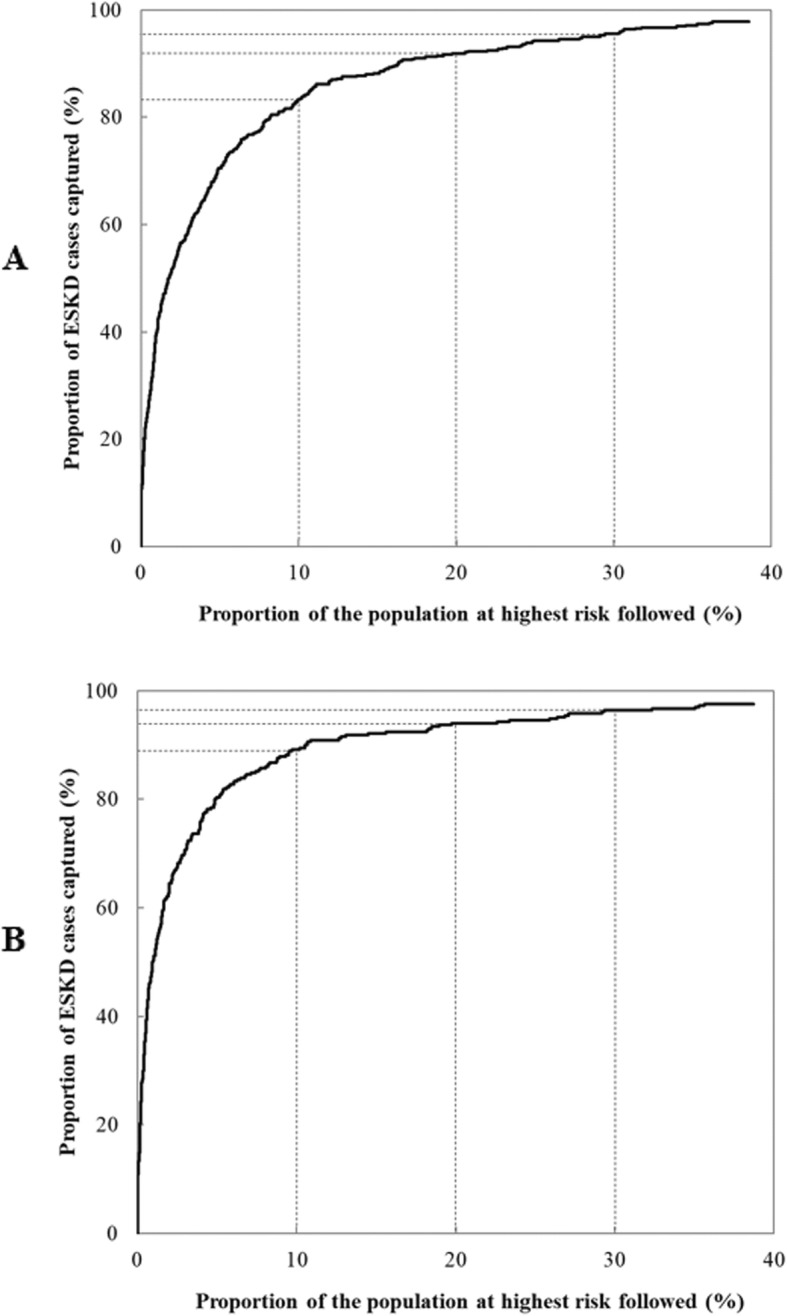
The proportion of cases followed and proportion of the population needed to be followed for the Recalibrated Pooled KFRE SEA equation for 2-year and 5-year risk of end-stage kidney disease. Legend: The figure shows the proportion of cases followed (y-axis) and proportion of the population needed to be followed (x-axis) for the Recalibrated Pooled KFRE SEA equation for **a**) 5-year risk and **b**) 2-year of end-stage kidney disease. The proportion of cases followed represents the proportion of individuals who will develop disease who are included in the proportion p of individuals in the population of the highest risk, and the proportion of the population needed to be followed is the proportion of the general population at highest risk that one needs to follow in order that a proportion q of those destined to become cases will be followed. At 5 years, an estimated 82, 92 and 96% events were captured in 10, 20 and 30% of subjects at the highest estimated risk of ESKD. At 2 years an estimated 89, 94 and 96% events were captured in 10, 20 and 30% of subjects at the highest estimated risk of ESKD. Abbreviation: ESKD, end-stage kidney disease; KFRE, Kidney Failure Risk Equation; SEA, Southeast Asia

Using the Recalibrated Pooled KFRE SEA at 5 years, an estimated 82% ESKD events were included among 10% of subjects at highest estimated risk of ESKD (Fig. 3), and an estimated 92 and 96% cases were included among 20 and 30% of subjects at highest ESKD risks (Fig. 3). At 2 years, an estimated 89, 94 and 96% events were captured in 10, 20 and 30% of subjects at the highest estimated risk of ESKD (Fig. 3).

**Fig. 4:**
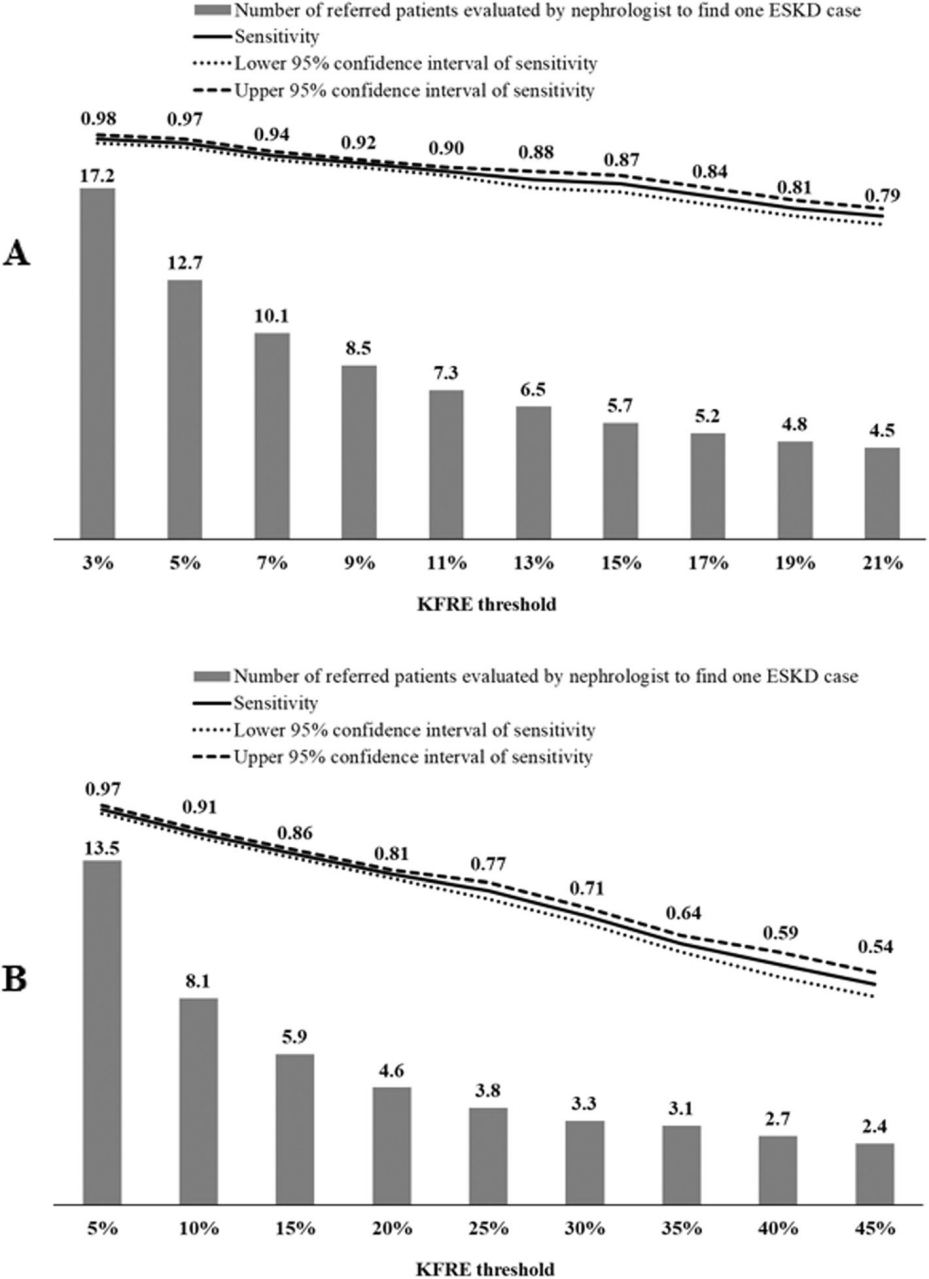
Number of patients identified as requiring referral to a nephrologist to find one ESKD case and sensitivity associated with a range of thresholds of the Recalibrated Pooled KFRE SEA equation for 5-year and 2-year risk of end-stage kidney disease. The figure shows number of patients identified as requiring referral to a nephrologist to find one ESKD case and sensitivity associated with a range of thresholds of Recalibrated Pooled KFRE SEA equation for **a**) 5-year and **b**) 2-year risk of end-stage kidney disease applied on the primary care patients with CKD from nine primary care clinics. The grey bar represents number of patients identified as requiring referral using each KFRE threshold as referral decision point to find one ESKD case, the solid line represents sensitivity of each KFRE threshold, and the dotted lines represent the upper and lower bound of 95% confidence interval of sensitivity. The Recalibrated Pooled KFRE SEA equation for 5-year ESKD risk was calculated as: 1–0.8362 ^ exp. (− 0.2245 × (age/10–7.036) + 0.3212 × (male - 0.5642) - 0.4553 × (eGFR/5–7.222) + 0.4469 × (lnACR - 5.137)). The Recalibrated Pooled KFRE SEA equation for 2-year ESKD risk was calculated as: 1–0.8976 ^ exp. (− 0.2245 × (age/10–7.036) + 0.3212 × (male - 0.5642) - 0.4553 × (eGFR/5–7.222) + 0.4469 × (lnACR - 5.137)). Abbreviation: ACR; albumin-to-creatinine ratio; CKD, chronic kidney disease; eGFR, estimated glomerular filtration rate; ESKD, end-stage kidney disease; KFRE, Kidney Failure Risk Equation; SEA, Southeast Asia

We also presented detailed statistics of a wide range of KFRE thresholds (3–21% at 5 year; 5–45% at 2 years) and observed that although using a higher KFRE threshold would refer fewer patients to a nephrologist to find one ESKD case, the sensitivity associated with KFRE also became less optimal (Fig. 4).

### Stratified and sensitivity analyses

KFRE discrimination remained excellent across all subgroups defined by 1) age (40–75 vs. > 75 years), 2) gender, 3) ethnicity (Chinese, Malays, and Indians), 4) type 2 diabetes mellitus status, and 5) CKD stages at both 5- and 2-year risks. At 5 years, the AUC ranged from 0.82 to 0.96 in the subgroups and at 2 years, the AUC ranged from 0.83 to 0.98 in the subgroups (Additional file 9). Of note, at 5 years, the 95% CI of Malays and Chinese were comparable; and that of Indians, maybe due to smaller sample sizes, was much wider than Chinese and Malays (Additional file 9).

A total of 408 and 236 ESKD events started dialysis and received kidney transplantation at 5-year and 2-year, respectively. Limiting to these events as outcomes, the discrimination of the Recalibrated Pooled KFRE SEA was largely the same as the main analyses: the AUC was 0.93 (95% CI: 0.92–0.94) at 5-year and 0.95 (95% CI: 0.93–0.96) at 2-year risk of ESKD onset.

A total of 3241 and 1927 deaths occurred during the 5-year and 2-year follow-ups, respectively. The competing risk and non-competing risk models at 5 years had similar HRs and considerate overlaping 95% CIs (242 [60.4–972] vs. 227 [56.5–909]), suggesting the effect of competing mortality risks is unlikely to affect the ESKD prediction.

## Discussion

### Statement of principal findings

Using electronic health records linked with national renal registry, we found that the recalibrated KFRE (Recalibrated Pooled KFRE SEA equation) had better performance than existing KFRE equations in terms of having a lower Brier score, less bias and improved precision for predicting ESKD in multi-ethnic patients visiting the primary care clinics. The overall predictive capability of the Recalibrated Pooled KFRE SEA for ESKD was significantly higher than using eGFR alone. In addition, 5-year KFRE thresholds ranging 10–16% for nephrologist referral and 2-year KFRE risk threshold at 45% for dialysis planning resulted in high referral efficiency, and substantially improved reclassification of ESKD risks relative to eGFR thresholds of 20, 30, 40 and 45 mL/min/1.73m^2^. Thus, automated referrals using KFRE thresholds warrant consideration in clinical practice for patients with CKD.

### In relation to previous studies

Previous studies showed excellent predictive utility of the Original KFRE equation or the Pooled KFRE equation primarily among patients with European origins or those in nephrology clinics [16–22]. Our study expands on those findings in the primary care clinics in SEA. We observed high AUCs of the Recalibrated Pooled KFRE SEA at both 5-year (0.94; 95% CI: 0.93–0.95) and 2-year (0.96; 95% CI: 0.95–0.97) risks of ESKD. Moreover, evidence for KFRE thresholds based on empirical data is limited and previous suggestions on KFRE thresholds have been based on physicians’ opinions [22]. Our study fills that gap by using comprehensive statistical metrics coupled with clinical consideration of nephrologist workload. Of note, our results suggested that the Recalibrated Pooled KFRE SEA thresholds ranging 10–16% for nephrology referral criterion over 5 years had high sensitivity, high specificity and high referral efficiency, and substantially improved reclassification of ESKD risk on top of eGFR thresholds of 30, 40 and 45 mL/min/1.73m^2^. In addition, the 2-year threshold of Recalibrated Pooled KFRE SEA for dialysis planning in the current study (45%) was close to the KFRE > 40% suggested previously [17] and was marginally better than eGFR 20 mL/min/1.73m^2^ [42]. Moreover, as the availabilities of the healthcare resources and the balance among sensitivity, specificity and referral efficiency may vary from country to country, a universal optimal KFRE threshold may not be possible. Our study provides a wide range of Recalibrated Pooled KFRE SEA thresholds with useful statistics (sensitivity and referral efficiency) for clinicians and health planners to choose from based on local resources, which greatly enhanced the clinical application to the primary care settings with different availability of nephrology resources globally.

### Meaning of the study

The Recalibrated Pooled KFRE SEA equation includes four routinely measured variables, which were available in > 86% of our study population with stage 3–5 CKD (although ACR was converted from urine albumin for all patients). Thus, our findings imply that the Recalibrated Pooled KFRE SEA equation is likely to aid referral decisions and dialysis planning across all general practitioner settings if integrated into EHR. Furthermore, the improved triage efficiency would enable patients at high-risk of ESKD to receive timely referrals to a nephrologist, which has been shown to shorten waiting time for nephrology care [46] and substantially reduce medical costs for initiating renal replacement therapy and dialysis compared to late referral [5]. Since the nephrologist shortage is global [10], the implications of shortening patient wait time and reducing costs would have significant impact on health systems and patient well-being in resource-limited settings where CKD burden is rising, and accessibility to renal replacement therapy is limited at a global level [47].

### Strengths and limitations

Our study has several strengths. First, this is likely to be the first report to determine the best-calibrated KFRE equation and potentially useful thresholds for nephrologist referral and dialysis planning in primary care population in SEA. When looking for the clinically useful thresholds, we applied rigorous statistical criteria, and combined service-related considerations for health planners, and the methodology provided as a yardstick for future studies. However, whehter the suggested thresholds are optimal for Singapore will need to be further tested taking into consideration of the workforce and work capacticy of Singapore nephrologists, and simulation studies are warranted to predict the performance of such thresholds over time. Second, we included all eligible patients visiting primary care clinics over the study duration and thus had a large sample size. Third, the multiple major ethnic groups in our sample (Chinese, Indians, and Malays) are a diaspora of populations from countries (China, India and Malaysia) that are homes to one-third of the world’s population. The excellent predictive utility of the Recalibrated Pooled KFRE SEA in all three ethnic groups shown in stratified analyses greatly enhanced the utilization of KFRE to many people globally. Fourth, serum creatinine measurements were calibrated to be traceable to an IDMS standard, thus increasing the validity of both eGFR and KFRE assessments. Fifth, we objectively assessed all ESKD cases with virtual follow-up completion via linkage to the nationwide Singapore Renal Registry.

However, our study also had some limitations. First, we deleted those without established care from the study (~ 30%), thus introducing the possibility of selection bias. Nevertheless, the prevalence of hypertension and diabetes was lower in the excluded population than that expected in the age-matched general population in Singapore [28, 48]. Second, the definition of ESKD in the current study was slightly different from that of Tangri et al. [16]; however, we conducted sensitivity analysis using the definition of ESKD from that of Tangri and found similar results. Third, the ACR value used in the KFRE score was converted from urine dipstick that was measured in all patients, and thus may be less precise compared to direct measurements. Specifically, the conversion between urine albumin to ACR for people with “+”, “++” and “+++” were based on limited data [17, 34, 35]. However, previous studies using the same conversion were included in the meta-analysis of KFRE validation and showed similar results of ESKD prediction [17], and thus suggested that the dipstick-converted ACR value is unlikely to have a large impact on the predictive performance of KFRE. Fourth, the current study did not have a validation dataset to examine the superior performance of Recalibrated KFRE SEA over other KFREs, and future studies among SEA populations are warranted to validate our results. Fifth, the sample size of Indian patients were small in the current population, and the 95% CI of AUC was wider compared to Chinese and Malays; thus, our results may not be generalizable to Indians. Future studies with bigger sample sizes of Indians are warranted to validate our results. Thus, the optimal threshold may be different in other countries. In addition, the current study did not have data on serum calcium, phosphate, bicarbonate, and albumin to validate the 8-variable KFRE equation. However, the 4- and 8-variable equations showed similar discrimination in the original development cohort [16] and subsequent meta-analysis [17]. Therefore, the less complicated 4-variable KFRE may be a more convenient tool for clinical usage.

## Conclusions

In conclusion, our results showed that the Recalibrated Pooled KFRE SEA equation is an excellent predictive tool and performed better in terms of having a lower Brier score, less bias and improved precision than existing KFRE for identifying patients with CKD at risk for progression to ESKD in a primary care setting in SEA. Our findings suggest that implementation of the equation using 5-year thresholds > 10–16% to guide dialysis planning and 2-year threshold > 45% to guide nephrologist referral would facilitate more efficient and accurate risk stratification of patients at high risk of ESKD. Future studies are warranted to validate our findings, evaluate the clinical and cost effectiveness of a CKD model of care that integrates EHR and the KFRE in primary care settings serving Asians as well as globally.

## Supplementary information

**Additional file 1:** **Figure S1.** Existing and recalibrated Kidney Failure Risk Equation (KFRE) for predicting 5-year risk of end-stage kidney disease among patients with chronic kidney disease stage 3–5. The figure shows the existing and recalibrated KFREs for predicting 5-year risk of end-stage kidney disease among patients with chronic kidney disease stage 3–5.

**Additional file 2 :** **Figure S2.** Existing and recalibrated Kidney Failure Risk Equation (KFRE) for predicting 2-year risk of end-stage kidney disease among patients with chronic kidney disease stage 3–5. The figure shows the existing and recalibrated KFREs for predicting 2-year risk of end-stage kidney disease among patients with chronic kidney disease stage 3–5.

**Additional file 3: Figure S3.** Calibration (Brier score, bias and precision) plots of Pooled Kidney Failure Risk Equation Southeast Asia (KFRE SEA) with different constants for 5-year and 2-year risks of end-stage kidney disease. The figure shows the Brier score, bias and precision associated with different Recalibrated Pooled KFRE SEA constants at A) 5-year and B) 2-year risks of end-stage kidney disease to evaluate how closely the predicted risks agree with the observed risks.

**Additional file 4: Table S1.** Baseline characteristics of patients with chronic kidney disease stage 3–5 with 2-year follow-up. The table shows baseline characteristics of patients with chronic kidney disease stage 3–5 included in the cohort with 2-year follow-up.

**Additional file 5: Figure S4.** Observed risk versus predicted probability of end-stage kidney disease using the Pooled Kidney Failure Risk Equation Southeast Asia (KFRE SEA) at five and 2 years. The predicted and observed end-stage kidney disease probability estimates represent the mean values of predicted risk and observed probabilities in the risk categories according to the Recalibrated Pooled KFRE SEA risks at A) 5-year and B) 2-year risks of end-stage kidney disease.

**Additional file 6: Figure S5.** Area under receiver operating characteristic curves of the Pooled Kidney Failure Risk Equation Southeast Asia (KFRE SEA) for predicting the 5- and 2-year risks of onset of end-stage kidney disease. The figure shows the Recalibrated Pooled KFRE SEA equations and eGFR receiver operating characteristic curves for predicting the A) 5-year and B) 2-year risks of end-stage kidney disease among patients with chronic kidney disease.

**Additional file 7: Table S2.** Reclassification of 5-year risk of end-stage kidney disease onset among chronic kidney disease patients using the Recalibrated Pooled Kidney Failure Risk Equation Southeast Asia (KFRE SEA) equation thresholds at 10 and 16% compared to estimated glomerular filtration rate 30, 40 and 45 mL/min/1.73m^2^. The table shows the net reclassification improvement of the Recalibrated Pooled KFRE SEA equation threshold at 10 and 16% compared to estimated glomerular filtration rate 30, 40 and 45 mL/min/1.73m^2^ for predicting the 5-year risk of end-stage kidney disease among patients with chronic kidney disease.

**Additional file 8: Table S3.** Reclassification of 2-year risk of end-stage kidney disease onset among chronic kidney disease patients using the Pooled Kidney Failure Risk Equation Southeast Asia (KFRE SEA) equation threshold at 45% compared to estimated glomerular filtration rate 20 and 18.8 mL/min/1.73m^2^. The table shows the net reclassification improvement of the Recalibrated Pooled KFRE SEA equation threshold at 45% compared to estimated glomerular filtration rate 20 and 18.8 mL/min/1.73m^2^ for predicting the 2-year risk of end-stage kidney disease among patients with chronic kidney disease.

**Additional file 9: Figure S6.** Stratified analyses of area under receiver operating characteristic curves of the Pooled Kidney Failure Risk Equation Southeast Asia (KFRE SEA) for predicting the 5-year and 2-year risks of end-stage kidney disease. The figure shows stratified analyses of area under receiver operating characteristic curves and 95% confidence interval of the Recalibrated Pooled KFRE SEA equations for predicting the A) 5-year and B) 2-year risk of onset of end-stage kidney disease.

## Data Availability

Data are available on reasonable request from the corresponding author subject to approval by the IRB.

## Abbreviations

ACR: Albumin-to-creatinine ratio
AUC: Area under receiver operating characteristic curve
CKD: Chronic kidney disease
CVD: Cardiovascular disease
eGFR: Estimated glomerular filtration rate
EHR: Electronic health records
ESKD: End-stage kidney disease
IDMS: Isotope dilution mass spectrometry
KFRE: Kidney Failure Risk Equation
LR-: Negative likelihood ratio
LR +: Positive likelihood ratio.
NPV: Negative predictive value
NRI: Net reclassification improvement
PPV: Positive predictive value
SEA: Southeast Asia
