# Supplementary material for: Validation of the kidney failure risk equation for end-stage kidney disease in Southeast Asia
Source: BMC Nephrol. 2019 Dec 4;20:451. doi: 10.1186/s12882-019-1643-0 (PMC6894117; doi:10.1186/s12882-019-1643-0)
Supplement: Supplementary file 1 — Additional file 1: Figure S1. Existing and recalibrated Kidney Failure Risk Equation (KFRE) for predicting 5-year risk of end-stage kidney disease among patients with chronic kidney disease stage 3–5. The figure shows the existing and recalibrated KFREs for predicting 5-year risk of end-stage kidney disease among patients with chronic kidney disease stage 3–5. [file 12882_2019_1643_MOESM1_ESM.docx]

**Additional file 1:**

**Supplemental Figure S1.** Existing and recalibrated Kidney Failure Risk Equation (KFRE) for predicting 5-year risk of end-stage kidney disease among patients with chronic kidney disease stage 3-5

**Existing KFRE equations, patient 5-year risk:**

*Original KFRE* [17]

1 – 0.9240 ^ exp (-0.2201 × (age/10 – 7.036) + 0.2467 × (male – 0.5642) – 0.5567 × (eGFR/5 – 7.222) + 0.4510 × (logACR – 5.137))

*Original KFRE Calibrated for North American* [17]

1 – 0.8996 ^ exp (-0.2201 × (age/10 – 7.036) + 0.2467 × (male – 0.5642) – 0.5567 × (eGFR/5 – 7.222) + 0.4510 × (logACR – 5.137))

*Original KFRE Calibrated for non-North American* [17]

1 – 0.9365 ^ exp (-0.2201 × (age/10 – 7.036) + 0.2467 × (male – 0.5642) – 0.5567 × (eGFR/5 – 7.222) + 0.4510 × (logACR – 5.137))

*Pooled KFRE* [17]

1 – 0.8762 ^ exp (-0.2245 × (age/10 – 7.036) + 0.3212 × (male – 0.5642) – 0.4553 × (eGFR/5 – 7.222) + 0.4469 × (logACR – 5.137))

**Modified Original KFRE equation, patient 5-year risk:**

*Modified Original KFRE SEA 1*

1 – 0.9595 ^ exp (-0.2201 × (age/10 – 7.036) + 0.2467 × (male – 0.5642) – 0.5567 × (eGFR/5 – 7.222) + 0.4510 × (logACR – 5.137))

*Modified Original KFRE SEA 2*

1 – 0.9595 ^ exp (-0.4734 × (age/10 – 7.036) + 0.0119 × (male – 0.5642) – 0.6990 × (eGFR/5 – 7.222) + 0.6159 × (logACR – 5.137))

**Modified Pooled KFRE equation, patient 5-year risk (the best-calibrated equation for the current population):**

*Modified Pooled KFRE SEA*

1 – 0.8362 ^ exp (-0.2245 × (age/10 – 7.036) + 0.3212 × (male – 0.5642) – 0.4553 × (eGFR/5 – 7.222) + 0.4469 × (logACR – 5.137))
