# Supplementary material for: Validation of the kidney failure risk equation for end-stage kidney disease in Southeast Asia
Source: BMC Nephrol. 2019 Dec 4;20:451. doi: 10.1186/s12882-019-1643-0 (PMC6894117; doi:10.1186/s12882-019-1643-0)
Supplement: Supplementary file 3 — Additional file 3: Figure S3. Calibration (Brier score, bias and precision) plots of Pooled Kidney Failure Risk Equation Southeast Asia (KFRE SEA) with different constants for 5-year and 2-year risks of end-stage kidney disease. The figure shows the Brier score, bias and precision associated with different Recalibrated Pooled KFRE SEA constants at A) 5-year and B) 2-year risks of end-stage kidney disease to evaluate how closely the predicted risks agree with the observed risks. [file 12882_2019_1643_MOESM3_ESM.docx]

**Additional file 3:**

**Supplemental Figure S3.** Calibration (Brier score, bias and precision) plots of Pooled Kidney Failure Risk Equation Southeast Asia (KFRE SEA) with different constants for 5-year and 2-year risks of end-stage kidney disease


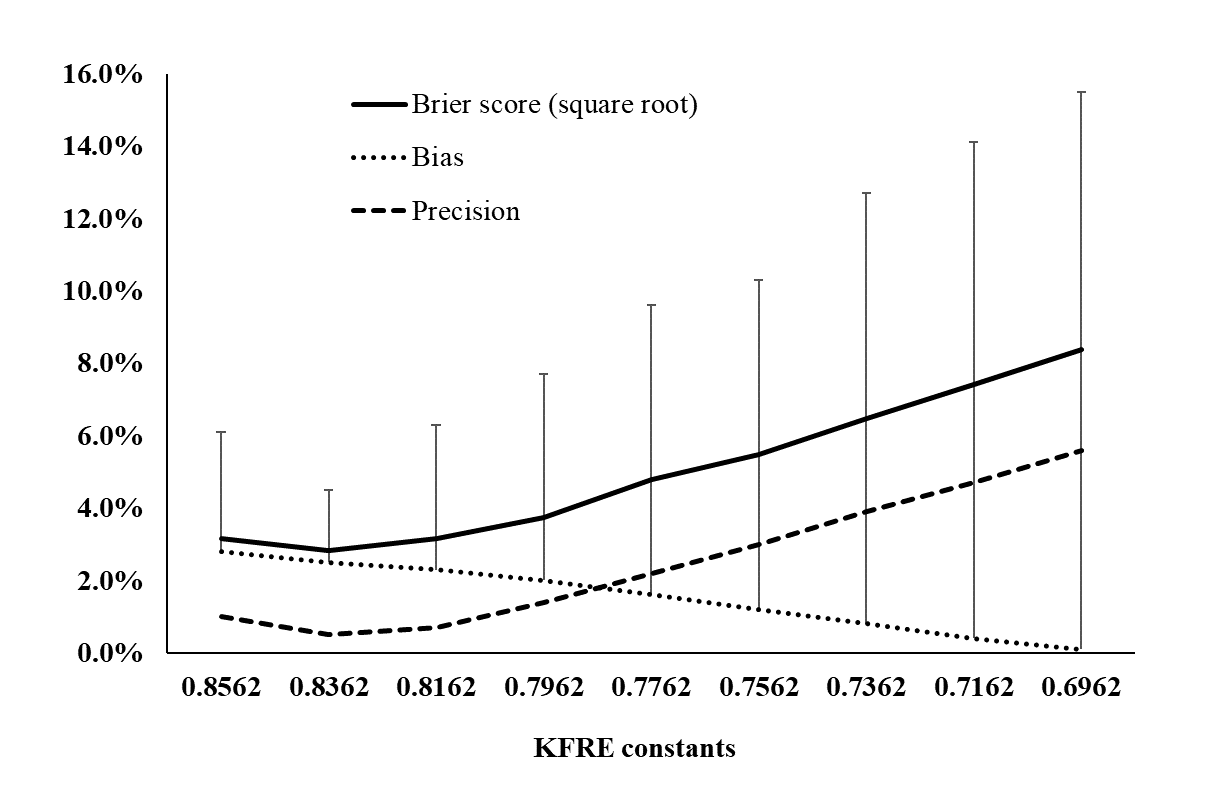


**A**

**
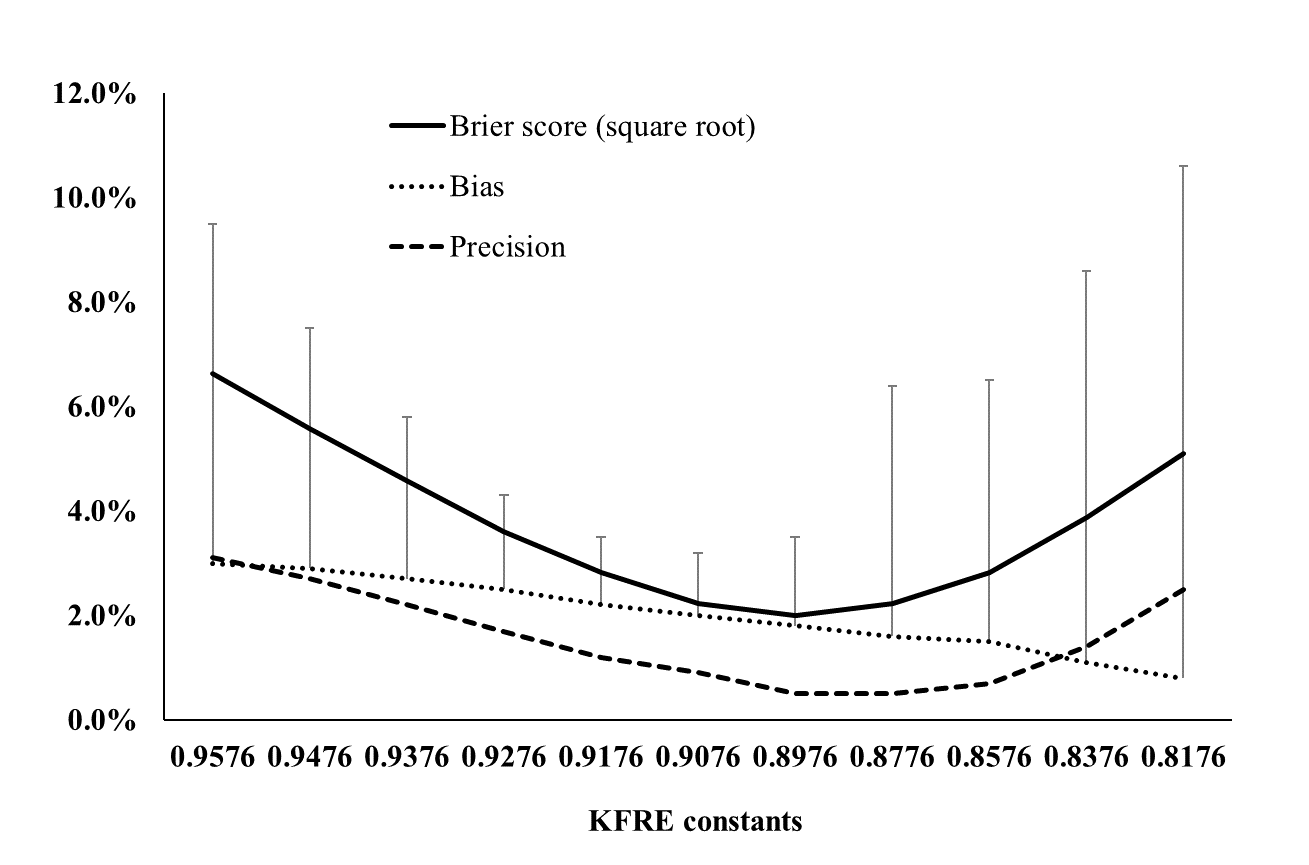
**

**B**

**Figure legend:** The figure shows the Brier score, bias and precision associated with different Recalibrated Pooled KFRE SEA constants at A) 5-year and B) 2-year risks of end-stage kidney disease to evaluate how closely the predicted risks agree with the observed risks. The Brier score calculates the squared difference of mean observed minus mean predicted risk, the bias calculates the median observed minus predicted risks and the precision is the interquartile range of the bias. A constant with the lowest scores of all three metrics would be chosen to constitute the KFRE equation with the best calibration. At five years, KFRE constant of 0.8362 had the lowest Brier score, the best precision, and the narrowest 95% confidence interval associated with the bias. At two years, KFRE constant of 0.8976 had the lowest Brier score, the best precision, and the second narrowest 95% confidence interval associated with the bias. The Recalibrated Pooled KFRE SEA equation for 5-year ESKD risk was calculated as: 1 - 0.8362 ^ exp (-0.2245 × (age/10 - 7.036) + 0.3212 × (male - 0.5642) - 0.4553 × (eGFR/5 - 7.222) + 0.4469 × (lnACR - 5.137)). The Recalibrated Pooled KFRE SEA equation for 2-year ESKD risk was calculated as: 1 - 0.8976 ^ exp (-0.2245 × (age/10 - 7.036) + 0.3212 × (male - 0.5642) - 0.4553 × (eGFR/5 - 7.222) + 0.4469 × (lnACR - 5.137)).

**Abbreviation:** ACR; albumin-to-creatinine ratio; eGFR, estimated glomerular filtration rate; ESKD, end-stage kidney disease; KFRE, Kidney Failure Risk Equation.
