# Supplementary material for: Validation of the kidney failure risk equation for end-stage kidney disease in Southeast Asia
Source: BMC Nephrol. 2019 Dec 4;20:451. doi: 10.1186/s12882-019-1643-0 (PMC6894117; doi:10.1186/s12882-019-1643-0)
Supplement: Supplementary file 4 — Additional file 4: Table S1. Baseline characteristics of patients with chronic kidney disease stage 3–5 with 2-year follow-up. The table shows baseline characteristics of patients with chronic kidney disease stage 3–5 included in the cohort with 2-year follow-up. [file 12882_2019_1643_MOESM4_ESM.docx]

**Additional file 4:**

**Supplemental Table S1.** Baseline characteristics of patients with chronic kidney disease stage 3-5 with 2-year follow-up^a^

| Variable | Total patients  with CKD  (*n* =17,444) | Patients with  ESKD  (*n* =330) | Patients without  ESKD  (*n* =17,114) |
| --- | --- | --- | --- |
| CKD stages, *n* (%) |  |  |  |
| Stage 3 CKD (30≤eGFR<60 mL/min/1.73m^2^) | 15,295 (88) | 48 (15) | 15,247 (89) |
| Stage 4 CKD (15≤eGFR<30 mL/min/1.73m^2^) | 1,976 (11) | 188 (57) | 1,788 (10) |
| Stage 5 CKD (eGFR<15 mL/min/1.73m^2^) | 173 (0.99) | 94 (28) | 79 (0.46) |
| Demographics |  |  |  |
| Age (years), mean (SD) | 76 (9) | 70 (11) | 76 (9) |
| Gender, *n* (%) |  |  |  |
| Men | 8,671 (50) | 176 (53) | 8,495 (50) |
| Women | 8,773 (50) | 154 (47) | 8,619 (50) |
| Ethnicity, *n* (%) |  |  |  |
| Chinese | 13,842 (79) | 239 (72) | 13,603 (80) |
| Indians | 731 (4) | 11 (3) | 720 (4) |
| Malays | 2,307 (13) | 68 (21) | 2,239 (13) |
| Others | 564 (3) | 12 (4) | 552 (3) |
| Lifestyle factors |  |  |  |
| Government housing, *n* (%) | 15,545 (89) | 303 (92) | 15,242 (89) |
| Past or current smoker, *n* (%) | 1,247 (7) | 31 (9) | 1,216 (7) |
| BMI (kg/m^2^), mean (SD) | 25.4 (4.54) | 26.0 (4.98) | 25.4 (4.53) |
| Known co-morbidities^b^ |  |  |  |
| Physician-diagnosed diabetes mellitus, *n* (%) | 10,282 (59) | 266 (81) | 10,016 (59) |
| Physician-diagnosed hypertension, *n* (%) | 17,135 (98) | 327 (99) | 16,808 (98) |
| Physician-diagnosed cardiovascular disease, *n* (%) | 4,411 (25) | 89 (27) | 4,322 (25) |
| Physician-diagnosed stroke, *n* (%) | 2,185 (13) | 44 (13) | 2,141 (13) |
| Recalibrated Pooled KFRE SEA equation^c^ |  |  |  |
| >20%, *n* (%) | 1,240 (7) | 268 (81) | 972 (6) |
| >40%, *n* (%) | 531 (3) | 195 (59) | 336 (2) |

^a^Data are expressed as mean (SD) for continuous variables and *n* (percentage) for categorical variables. CKD was defined as CKD-EPI eGFR <60 mL/min/1.73m^2^.

^b^Known co-morbidities as documented by physicians in the electronic health record.

^c^The Recalibrated Pooled KFRE SEA equation at 2-year ESKD risk was calculated as: 1 - 0.8976 ^ exp (-0.2245 × (age/10 - 7.036) + 0.3212 × (male - 0.5642) - 0.4553 × (eGFR/5 - 7.222) + 0.4469 × (lnACR - 5.137)).

**Abbreviation:** ACR, albumin-to-creatinine ratio; BMI, body mass index; CKD, chronic kidney disease; CKD-EPI, Chronic Kidney Disease Epidemiology Collaboration; eGFR, estimated glomerular filtration rate; ESKD, end-stage kidney disease; KFRE, Kidney Failure Risk Equation; SEA, Southeast Asia; SD, standard deviation.
