# Supplementary material for: Validation of the kidney failure risk equation for end-stage kidney disease in Southeast Asia
Source: BMC Nephrol. 2019 Dec 4;20:451. doi: 10.1186/s12882-019-1643-0 (PMC6894117; doi:10.1186/s12882-019-1643-0)
Supplement: Supplementary file 5 — Additional file 5: Figure S4. Observed risk versus predicted probability of end-stage kidney disease using the Pooled Kidney Failure Risk Equation Southeast Asia (KFRE SEA) at five and 2 years. The predicted and observed end-stage kidney disease probability estimates represent the mean values of predicted risk and observed probabilities in the risk categories according to the Recalibrated Pooled KFRE SEA risks at A) 5-year and B) 2-year risks of end-stage kidney disease. [file 12882_2019_1643_MOESM5_ESM.docx]

**Additional file 5:**

**Supplemental Figure S4.** Observed risk versus predicted probability of end-stage kidney disease using the Pooled Kidney Failure Risk Equation Southeast Asia (KFRE SEA) at five and two years


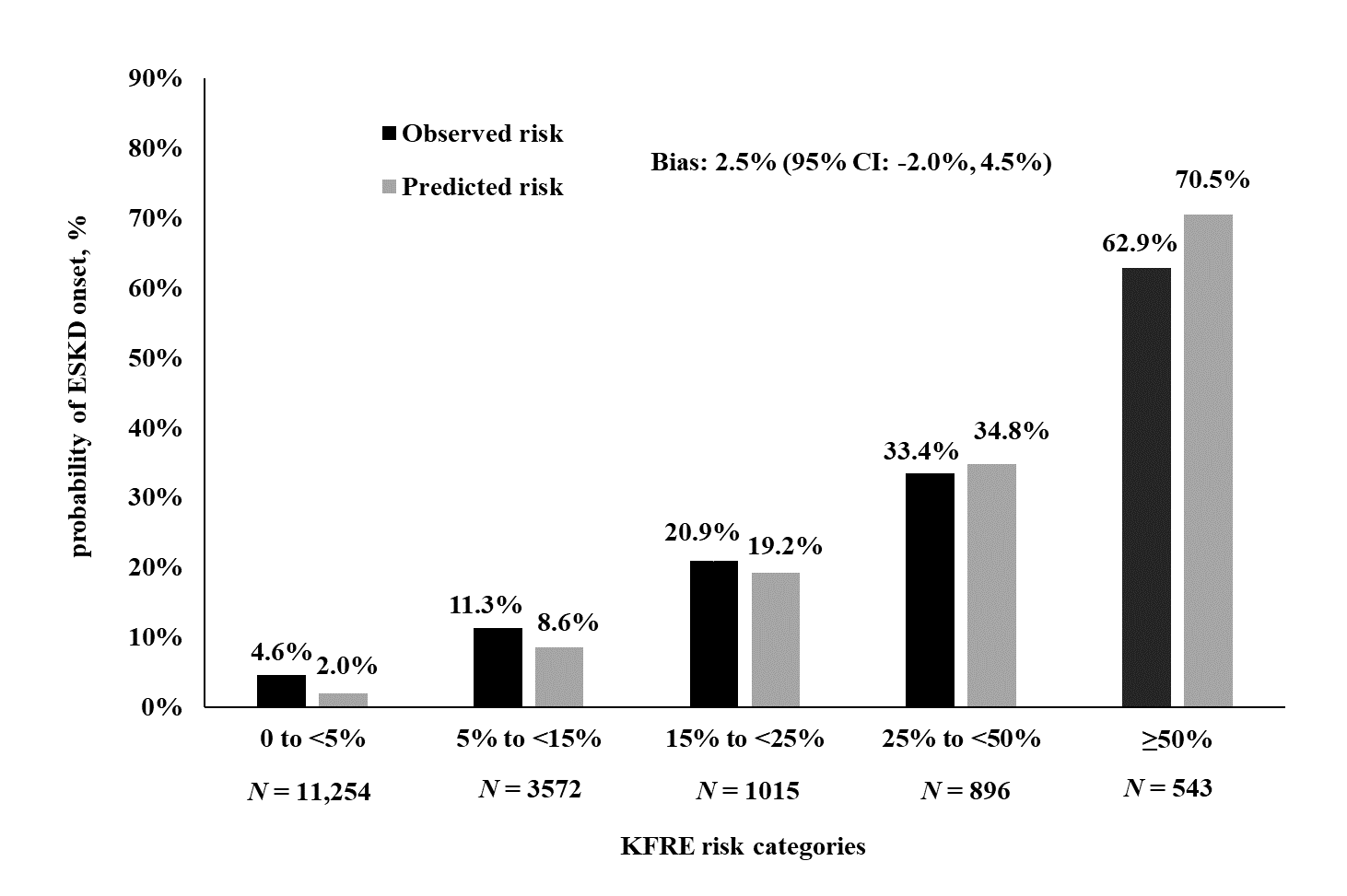


**A**


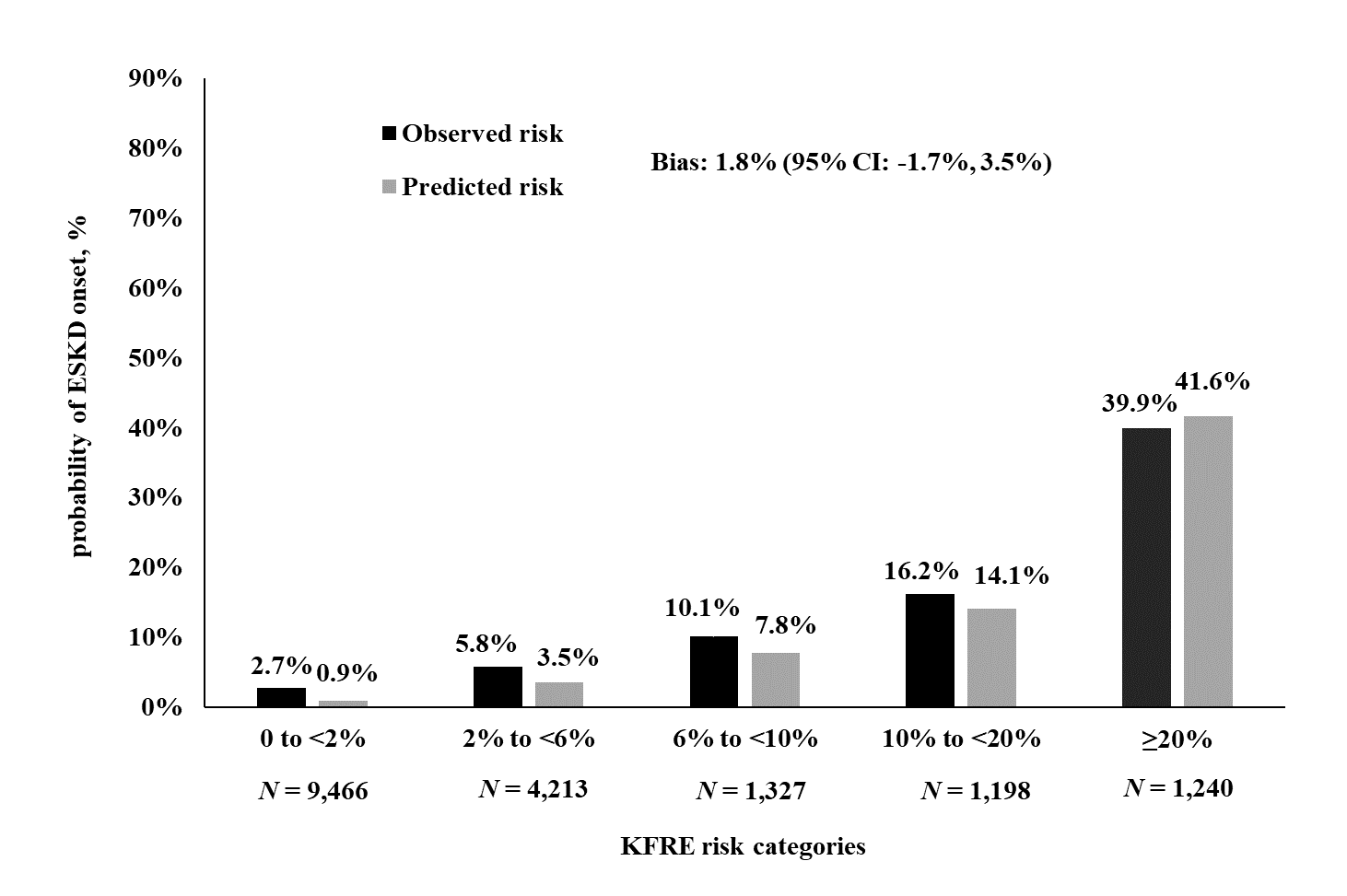


**B**

**Figure legend:** The predicted and observed end-stage kidney disease probability estimates represent the mean values of predicted risk and observed probabilities in the risk categories according to the Recalibrated Pooled KFRE SEA risks at A) 5-year and B) 2-year risks of end-stage kidney disease. The Recalibrated Pooled KFRE SEA equation at 5-year ESKD risk used for the current population was 1 - 0.8362 ^ exp (-0.2245 × (age/10 - 7.036) + 0.3212 × (male - 0.5642) - 0.4553 × (eGFR/5 - 7.222) + 0.4469 × (lnACR - 5.137)). The bias (median observed minus predicted risks) was 2.5% (95% CI: -2.0%-4.5%). The Recalibrated Pooled KFRE SEA equation at 2-year ESKD risk used for the current population was 1 - 0.8976 ^ exp (-0.2245 × (age/10 - 7.036) + 0.3212 × (male - 0.5642) - 0.4553 × (eGFR/5 - 7.222) + 0.4469 × (lnACR - 5.137)). The bias was 1.8% (95% CI: -1.7%-3.5%).

**Abbreviation:** ACR; albumin-to-creatinine ratio; CI, confidence interval; eGFR, estimated glomerular filtration rate; ESKD, end-stage kidney disease; KFRE, Kidney Failure Risk Equation; SEA, Southeast Asia.
