# Supplementary material for: Validation of the kidney failure risk equation for end-stage kidney disease in Southeast Asia
Source: BMC Nephrol. 2019 Dec 4;20:451. doi: 10.1186/s12882-019-1643-0 (PMC6894117; doi:10.1186/s12882-019-1643-0)
Supplement: Supplementary file 6 — Additional file 6: Figure S5. Area under receiver operating characteristic curves of the Pooled Kidney Failure Risk Equation Southeast Asia (KFRE SEA) for predicting the 5- and 2-year risks of onset of end-stage kidney disease. The figure shows the Recalibrated Pooled KFRE SEA equations and eGFR receiver operating characteristic curves for predicting the A) 5-year and B) 2-year risks of end-stage kidney disease among patients with chronic kidney disease. [file 12882_2019_1643_MOESM6_ESM.docx]

**Additional file 6:**

**Supplemental Figure S5.** Area under receiver operating characteristic curves of the Pooled Kidney Failure Risk Equation Southeast Asia (KFRE SEA) for predicting the 5- and 2-year risks of onset of end-stage kidney disease


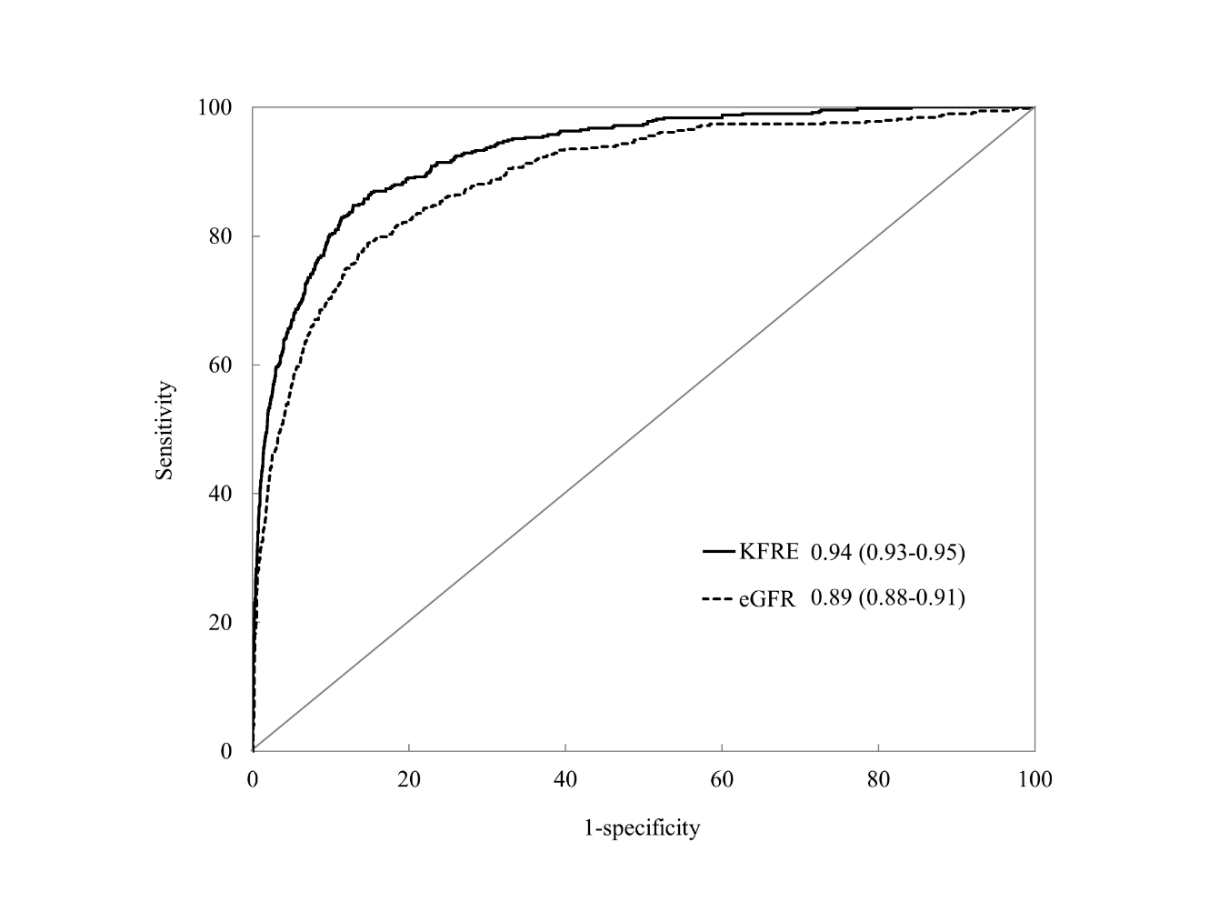


**A**

**
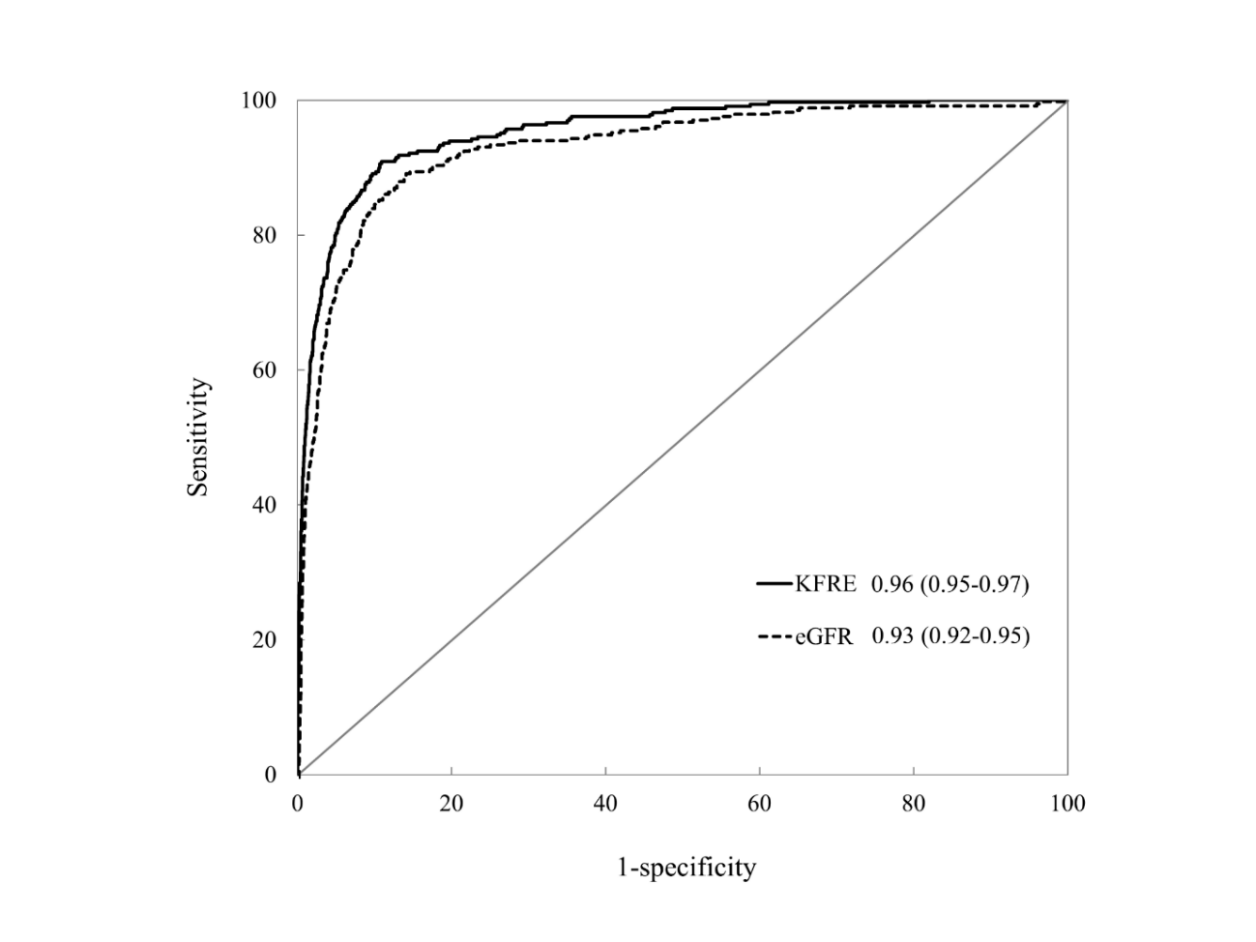
**

**B**

**Figure legend:** The figure shows the Recalibrated Pooled KFRE SEA equations and eGFR receiver operating characteristic curves for predicting the A) 5-year and B) 2-year risks of end-stage kidney disease among patients with chronic kidney disease. For the 5-year risk, area under receiver operating characteristic curve and 95% confidence intervals are 0.94 (0.93-0.95) and 0.89 (0.88-0.91), respectively. For the 2-year risk, area under receiver operating characteristic curve and 95% confidence intervals were 0.96 (0.95-0.97) and 0.93 (0.92-0.95), respectively.

**Abbreviations:** eGFR, estimated glomerular filtration rate; KFRE, Kidney Failure Risk Equation; SEA, Southeast Asia.
