# Supplementary material for: Validation of the kidney failure risk equation for end-stage kidney disease in Southeast Asia
Source: BMC Nephrol. 2019 Dec 4;20:451. doi: 10.1186/s12882-019-1643-0 (PMC6894117; doi:10.1186/s12882-019-1643-0)
Supplement: Supplementary file 7 — Additional file 7: Table S2. Reclassification of 5-year risk of end-stage kidney disease onset among chronic kidney disease patients using the Recalibrated Pooled Kidney Failure Risk Equation Southeast Asia (KFRE SEA) equation thresholds at 10 and 16% compared to estimated glomerular filtration rate 30, 40 and 45 mL/min/1.73m2. The table shows the net reclassification improvement of the Recalibrated Pooled KFRE SEA equation threshold at 10 and 16% compared to estimated glomerular filtration rate 30, 40 and 45 mL/min/1.73m2 for predicting the 5-year risk of end-stage kidney disease among patients with chronic kidney disease. [file 12882_2019_1643_MOESM7_ESM.docx]

**Additional file 7:**

**Supplemental Table S2.** Reclassification of 5-year risk of end-stage kidney disease onset among chronic kidney disease patients using the Pooled Kidney Failure Risk Equation Southeast Asia (KFRE SEA) equation thresholds at 10% and 16% compared to estimated glomerular filtration rate 30, 40 and 45 mL/min/1.73m^2a^

| Recalibrated Pooled KFRE SEA at 10% compared with eGFR 30 mL/min/1.73m^2^ | All | Assigned to higher ESKD risk | Assigned to lower ESKD risk | NRI | | |
| --- | --- | --- | --- | --- | --- | --- |
| Expected number, event patients | 491 | 108 | 4 |  | Among event patients | 21.2% |
| Expected number, non-event patients | 16,780 | 1,700 | 256 |  | Among non-event patients | -8.61% |
|  | | | | | Overall original (95% CI) | 12.6% (10.6%, 15.3%) |
| Recalibrated Pooled KFRE SEA at 10% compared with eGFR 35.4 mL/min/1.73m^2^ | All | Assigned to higher ESKD risk | Assigned to lower ESKD risk | NRI | | |
| Expected number, event patients | 491 | 52 | 7 |  | Among event patients | 9.16% |
| Expected number, non-event patients | 16,780 | 954 | 1,019 |  | Among non-event patients | 0.39% |
|  | | | | | Overall original (95% CI) | 9.55% (7.91%, 11.5%) |
| Recalibrated Pooled KFRE SEA at 10% compared with eGFR 40 mL/min/1.73m^2^ | All | Assigned to higher ESKD risk | Assigned to lower ESKD risk | NRI | | |
| Expected number, event patients | 491 | 25 | 10 |  | Among event patients | 3.05% |
| Expected number, non-event patients | 16,780 | 495 | 2,287 |  | Among non-event patients | 10.7% |
|  | | | | | Overall original (95% CI) | 13.8% (11.7%, 16.0%) |
| Recalibrated Pooled KFRE SEA at 10% compared with eGFR 45 mL/min/1.73m^2^ | All | Assigned to higher ESKD risk | Assigned to lower ESKD risk | NRI | | |
| Expected number, event patients | 491 | 9 | 22 |  | Among event patients | -2.65% |
| Expected number, non-event patients | 16,780 | 193 | 4,487 |  | Among non-event patients | 25.6% |
|  | | | | | Overall original (95% CI) | 23.0% (20.5%, 25.7%) |
| Recalibrated Pooled KFRE SEA at 16% compared with eGFR 30 mL/min/1.73m^2^ | All | Assigned to higher ESKD risk | Assigned to lower ESKD risk | NRI | | |
| Expected number, event patients | 491 | 90 | 11 |  | Among event patients | 16.1% |
| Expected number, non-event patients | 16,780 | 798 | 527 |  | Among non-event patients | -1.62% |
|  | | | | | Overall original (95% CI) | 14.5% (12.5%, 16.8%) |
| Recalibrated Pooled KFRE SEA at 16% compared with eGFR 40 mL/min/1.73m^2^ | All | Assigned to higher ESKD risk | Assigned to lower ESKD risk | NRI | | |
| Expected number, event patients | 491 | 14 | 24 |  | Among event patients | -2.04% |
| Expected number, non-event patients | 16,780 | 172 | 3,137 |  | Among non-event patients | 20.1% |
|  | | | | | Overall original (95% CI) | 18.1% (15.8%, 20.6%) |
| Recalibrated Pooled KFRE SEA at 16% compared with eGFR 45 mL/min/1.73m^2^ | All | Assigned to higher ESKD risk | Assigned to lower ESKD risk | NRI | | |
| Expected number, event patients | 491 | 4 | 42 |  | Among event patients | -7.74% |
| Expected number, non-event patients | 16,780 | 37 | 5,504 |  | Among non-event patients | 32.6% |
|  | | | | | Overall original (95% CI) | 24.8% (22.2%, 27.6%) |

^a^NRI assessed the addition of Recalibrated Pooled KFRE SEA equation to a base model including binary eGFR (< vs. ≥30 mL/min/1.73m^2^, < vs. ≥40 mL/min/1.73m^2^, and < vs. ≥45 mL/min/1.73m^2^). CKD was defined as CKD-EPI eGFR <60 mL/min/1.73m^2^. The optimal threshold identified by Youden Index was 16% for Recalibrated Pooled KFRE SEA for predicting 5-year ESKD risk. The Recalibrated Pooled KFRE SEA equation for 5-year ESKD risk was calculated as: 1 - 0.8362 ^ exp (-0.2245 × (age/10 - 7.036) + 0.3212 × (male - 0.5642) - 0.4553 × (eGFR/5 - 7.222) + 0.4469 × (lnACR - 5.137)).

**Abbreviations:** ACR; albumin-to-creatinine ratio; CKD, chronic kidney disease; CKD-EPI, Chronic Kidney Disease Epidemiology Collaboration; eGFR, estimated glomerular filtration rate; ESKD, end-stage kidney disease; KFRE, Kidney Failure Risk Equation; NRI, net reclassification improvement; SEA, Southeast Asia.
