# Supplementary material for: Validation of the kidney failure risk equation for end-stage kidney disease in Southeast Asia
Source: BMC Nephrol. 2019 Dec 4;20:451. doi: 10.1186/s12882-019-1643-0 (PMC6894117; doi:10.1186/s12882-019-1643-0)
Supplement: Supplementary file 8 — Additional file 8: Table S3. Reclassification of 2-year risk of end-stage kidney disease onset among chronic kidney disease patients using the Pooled Kidney Failure Risk Equation Southeast Asia (KFRE SEA) equation threshold at 45% compared to estimated glomerular filtration rate 20 and 18.8 mL/min/1.73m2. The table shows the net reclassification improvement of the Recalibrated Pooled KFRE SEA equation threshold at 45% compared to estimated glomerular filtration rate 20 and 18.8 mL/min/1.73m2 for predicting the 2-year risk of end-stage kidney disease among patients with chronic kidney disease. [file 12882_2019_1643_MOESM8_ESM.docx]

**Additional file 8:**

**Supplemental Table S3.** Reclassification of 2-year risk of end-stage kidney disease onset among chronic kidney disease patients using the Pooled Kidney Failure Risk Equation Southeast Asia (KFRE SEA) equation threshold at 45% compared to estimated glomerular filtration rate 20 and 78.8 mL/min/1.73m^2a^

| Recalibrated Pooled KFRE SEA at 45% compared with eGFR 20 mL/min/1.73m^2^ | All | | Assigned to higher ESKD risk | | Assigned to lower ESKD risk | | NRI | | | | | | |  |  |
| --- | --- | --- | --- | --- | --- | --- | --- | --- | --- | --- | --- | --- | --- | --- | --- |
| Expected number, event patients | 330 | | 47 | | 39 | |  | | Among event patients | | 2.42% | | |  |  |
| Expected number, non-event patients | 17,114 | | 110 | | 234 | |  | | Among non-event patients | | 0.72% | | |  |  |
|  | | | | | | | | | Overall original (95% CI) | | 3.14% (2.86%, 3.43%) | | |  |  |
| Recalibrated Pooled KFRE SEA at 45% compared with eGFR 18.8 mL/min/1.73m^2^ | | All | | Assigned to higher ESKD risk | | Assigned to lower ESKD risk | | NRI | | | | | |  |  |
| Expected number, event patients | | 330 | | 57 | | 34 | |  | | Among event patients | | | 6.97% | | |
| Expected number, non-event patients | | 17,114 | | 131 | | 157 | |  | | Among non-event patients | | | 0.09% | | |
|  | | | | | | | | | Overall original (95% CI) | | | 7.06% (6.77%, 7.34%) | | |  |

^a^NRI assessed the addition of the Recalibrated Pooled KFRE SEA equation to a base model including binary eGFR (< vs. ≥20 mL/min/1.73m^2^). CKD was defined as CKD-EPI eGFR <60 mL/min/1.73m^2^. The Recalibrated Pooled KFRE SEA equation for 2-year ESKD risk was calculated as: 1 - 0.8976 ^ exp (-0.2245 × (age/10 - 7.036) + 0.3212 × (male - 0.5642) - 0.4553 × (eGFR/5 - 7.222) + 0.4469 × (lnACR - 5.137)).

**Abbreviations:** ACR; albumin-to-creatinine ratio; CKD, chronic kidney disease; CKD-EPI, Chronic Kidney Disease Epidemiology Collaboration; eGFR, estimated glomerular filtration rate; ESKD, end-stage kidney disease; KFRE, Kidney Failure Risk Equation; NRI, net reclassification improvement; SEA, Southeast Asia.
