# Supplementary material for: Validation of the kidney failure risk equation for end-stage kidney disease in Southeast Asia
Source: BMC Nephrol. 2019 Dec 4;20:451. doi: 10.1186/s12882-019-1643-0 (PMC6894117; doi:10.1186/s12882-019-1643-0)
Supplement: Supplementary file 9 — Additional file 9: Figure S6. Stratified analyses of area under receiver operating characteristic curves of the Pooled Kidney Failure Risk Equation Southeast Asia (KFRE SEA) for predicting the 5-year and 2-year risks of end-stage kidney disease. The figure shows stratified analyses of area under receiver operating characteristic curves and 95% confidence interval of the Recalibrated Pooled KFRE SEA equations for predicting the A) 5-year and B) 2-year risk of onset of end-stage kidney disease. [file 12882_2019_1643_MOESM9_ESM.docx]

**Additional file 9:**

**Supplemental Figure S6.** Stratified analyses of area under receiver operating characteristic curves of the Pooled Kidney Failure Risk Equation Southeast Asia (KFRE SEA) for predicting the 5-year and 2-year risks of end-stage kidney disease

**A**


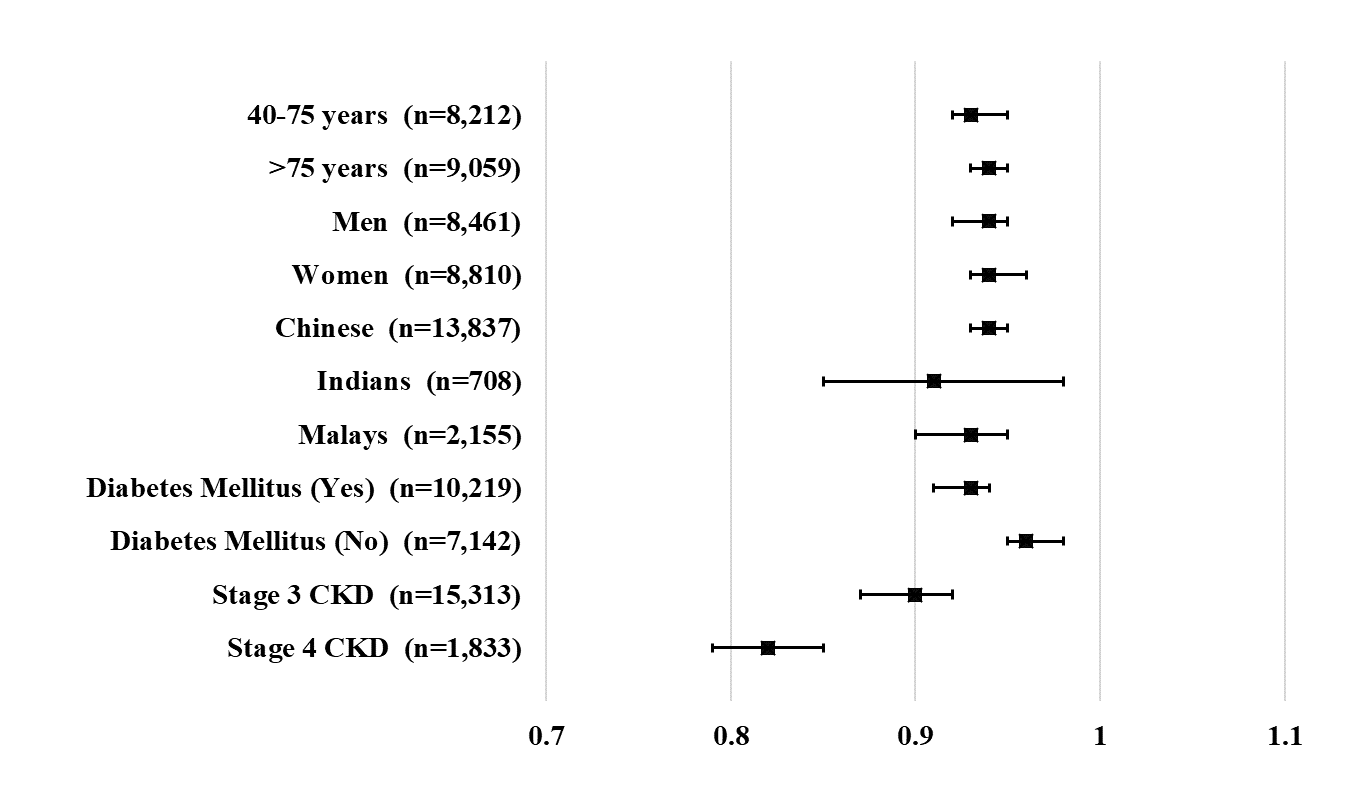


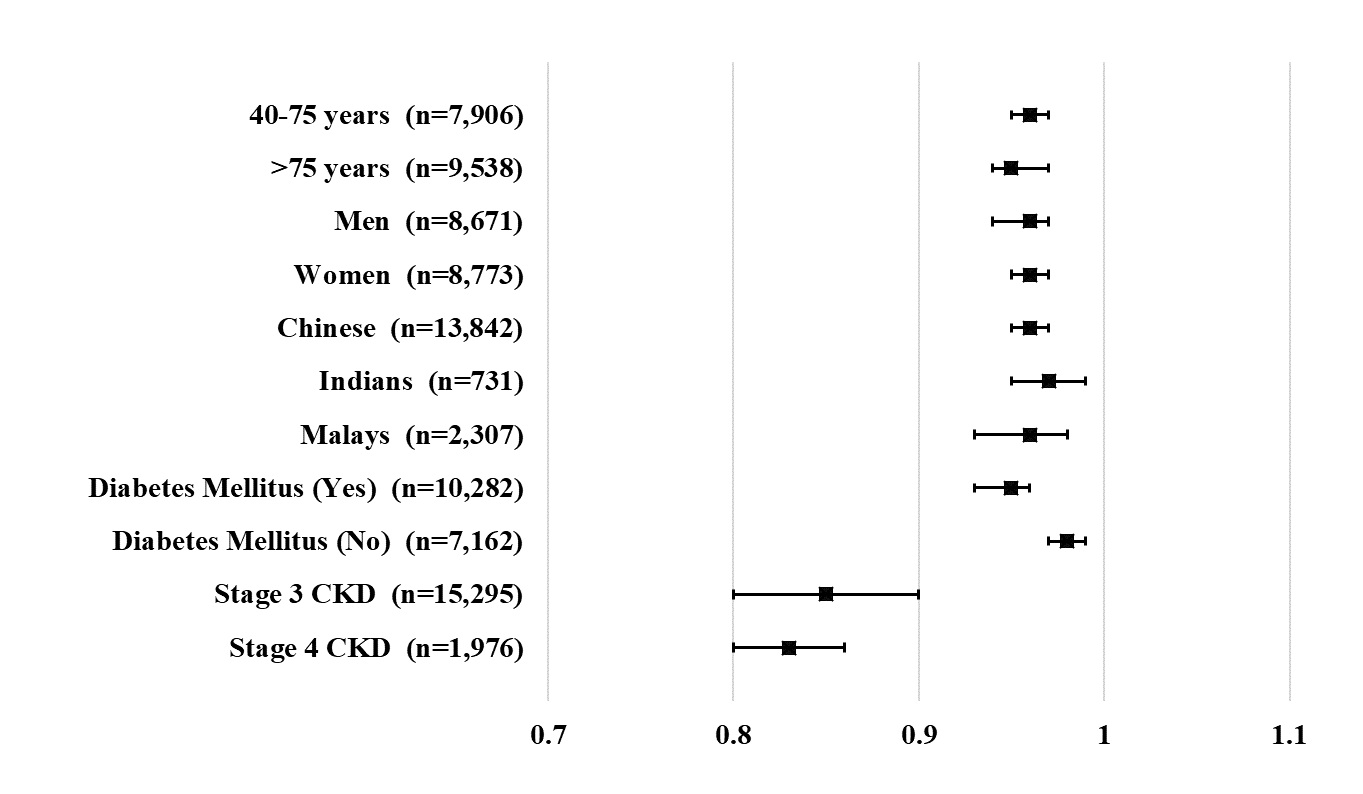


**B**

**Figure legend:** The figure shows stratified analyses of area under receiver operating characteristic curves and 95% confidence interval of the Recalibrated Pooled KFRE SEA equations for predicting the A) 5-year and B) 2-year risk of onset of end-stage kidney disease. Stage 3 CKD was defined as CKD-EPI eGFR ≥30 and <60 mL/min/1.73m^2^, and stage 4 CKD was defined as CKD-EPI eGFR ≥15 and <30 mL/min/1.73m^2^.

**Abbreviation:** CKD-EPI, Chronic Kidney Disease Epidemiology Collaboration; eGFR, estimated glomerular filtration rate; CKD, chronic kidney disease.
